# Mycolic acid-containing bacteria trigger distinct types of membrane vesicles through different routes

**DOI:** 10.1016/j.isci.2020.102015

**Published:** 2021-01-14

**Authors:** Toshiki Nagakubo, Yuhei O. Tahara, Makoto Miyata, Nobuhiko Nomura, Masanori Toyofuku

**Affiliations:** 1 Department of Life and Environmental Sciences, University of Tsukuba, Tsukuba, Japan; 2 Current affiliation: Graduate School of Agricultural and Life Sciences, The University of Tokyo, Tokyo, Japan; 3 Graduate School of Science, Osaka City University, Osaka, Japan; 4 The OCU Advanced Research Institute for Natural Science and Technology (OCARINA), Osaka City University, Osaka, Japan; 5 Microbiology Research Center for Sustainability (MiCS), University of Tsukuba, Tsukuba, Japan

**Keywords:** Microbiology, Cell Biology

## Abstract

Bacterial membrane vesicles (MVs) are attracting considerable attention in diverse fields of life science and biotechnology due to their potential for various applications. Although there has been progress in determining the mechanisms of MV formation in Gram-negative and Gram-positive bacteria, the mechanisms in mycolic acid-containing bacteria remain an unsolved question due to its complex cell envelope structure. Here, by adapting super-resolution live-cell imaging and biochemical analysis, we show that *Corynebacterium glutamicum* form distinct types of MVs via different routes in response to environmental conditions. DNA-damaging stress induced MV formation through prophage-triggered cell lysis, whereas envelope stress induced MV formation through mycomembrane blebbing. The MV formation routes were conserved in other mycolic acid-containing bacteria. Our results show how the complex cell envelope structure intrinsically generates various types of MVs and will advance our knowledge on how different types of MVs can be generated from a single cell organism.

## Highlights

• Distinct types of MVs are formed in bacteria with complex cell envelopes
• MVs can be formed from growing and dying cells via different routes
• Unique multivesicular MVs can be formed
• The MV formation routes are conserved among several mycolic-acid containing bacteria

## Graphical Abstract

**Figure:**
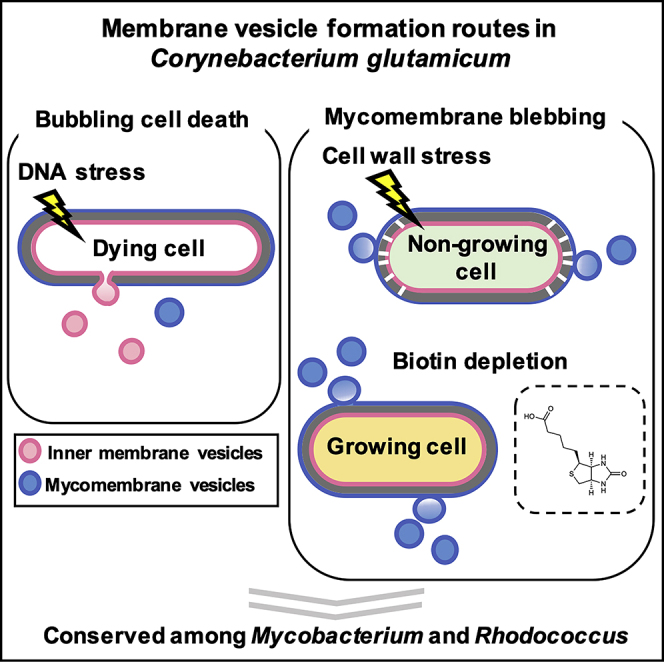

## Introduction

Most bacteria form membrane vesicles, which play important roles in various biological processes (Schwechheimer and Kuehn, 2015) such as bacterial communication (Toyofuku et al., 2017b), resistance to antibiotics and phages (Manning and Kuehn, 2011), and immunomodulation of the host (Vidakovics et al., 2010). Due to these various biological functions and their great potential for application in biotechnology, such as the development of vaccines (Robbins and Moreli, 2014) and drug delivery vehicles (Gujrati et al., 2014), MVs have been attracting attention of researchers in broad areas of life science and biotechnology and the understanding of MV formation mechanisms are fundamental. MVs were classically thought to be formed by the blebbing of the outer membrane in Gram-negative bacteria and therefore were called outer membrane vesicles (OMVs). Outer membrane blebbing is caused by either unbalanced cell envelope biosynthesis or the intercalation of hydrophobic molecules into the outer membrane (Schwechheimer and Kuehn, 2015). In addition, it was recently shown that a Gram-negative bacterium, *Pseudomonas aeruginosa*, can form MVs through explosive cell lysis that is triggered by the expression of peptidoglycan-degrading enzyme, endolysin, encoded in a cryptic phage region (Turnbull et al., 2016). Explosive cell lysis is a process where endolysin degrades the peptidoglycan, resulting in cells lysis with shattered cellular membrane fragments subsequently rounding up and forming MVs (Toyofuku et al., 2019; Turnbull et al., 2016). Endolysin also triggers MV formation in Gram-positive bacteria, such as *Bacillus subtilis* (Toyofuku et al., 2017a) and *Staphylococcus aureus* (Andreoni et al., 2019), but through a distinct process as the cell morphology stays intact due to the thick cell wall. This process is named bubbling cell death (Toyofuku et al., 2019) where endolysin initially forms holes in the cell wall through which membrane protrudes and forms cytoplasmic membrane vesicles (CMVs) (Toyofuku et al., 2019). In contrast to the progress made for Gram-negative and Gram-positive bacteria, the mechanism of MV formation in mycolic acid-containing bacteria (MCB) remains poorly understood.

**Figure 1:**
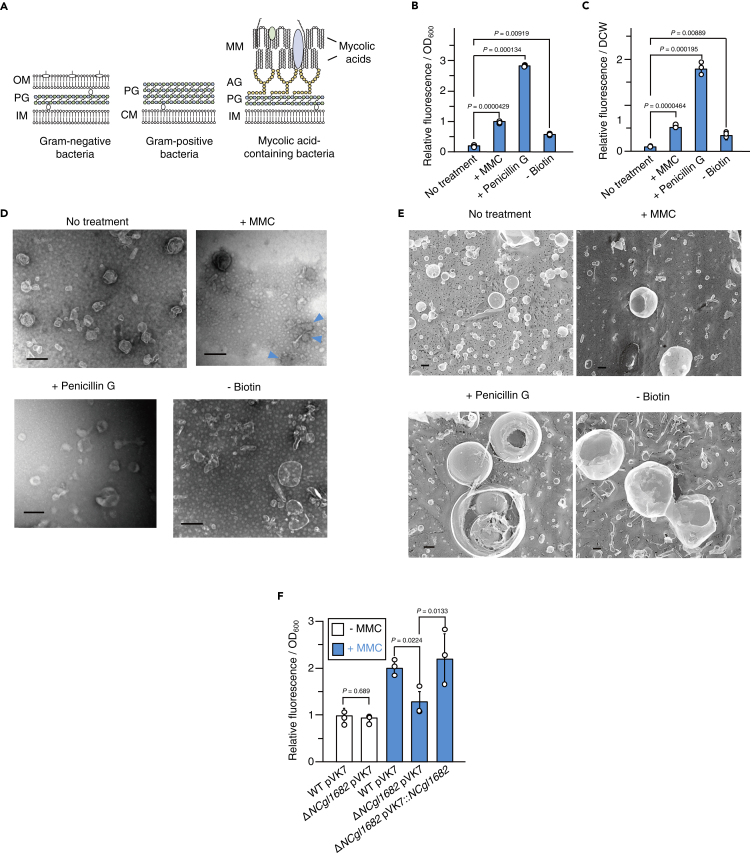
Induction of membrane vesicle formation in *Corynebacterium glutamicum* (A) Structures of the cell envelope of Gram-negative (left), Gram-positive (middle), and mycolic acid-containing bacteria (right). OM, outer membrane; MM, mycomembrane; PG, peptidoglycan; IM, inner membrane; and AG, arabinogalactan; CM, cytoplasmic membrane. (B and C) Membrane vesicle (MV) release by *C. glutamicum* under MV formation-inducing conditions. FM4-64 fluorescence of the purified MV fractions was normalized to (B) OD_600_ or (C) dried cell weight (DCW). All values indicated by the bars represent the mean value ± SD for three experiments. p values were calculated using unpaired t test with Welch's correction. (D) Transmission electron microscopic (TEM) images of MVs released by *C. glutamicum* under the conditions shown in (B and C). Scale bars, 200 nm. Structures that are presumably MVs collapsed are indicated with blue arrowheads. (E) Quick-freeze deep-etch (QFDE) electron microscopic images of MVs. Scale bars, 200 nm. (F) MV release by wild-type *C. glutamicum* and *NCgl1682* deletion mutant. White and blue bars indicate the presence or absence of MMC in the culture media, respectively. All values indicated by the bars represent the mean value ± SD for three experiments. p values were calculated using unpaired t test with Welch's correction.

A main reason why MV formation is not well understood in MCB is due to their complex cell structure. MCB include industrially and clinically important species of *Rhodococcus* and *Mycobacterium,* such as *Mycobacterium tuberculosis* (Prados-Rosales et al., 2011), and are characterized by their unique lipid-rich cell envelope structures. MCB possess a mycomembrane (Dulberger et al., 2020), outside of the thick cell wall, which mainly consists of mycolic acids (Figure 1A). The mycomembrane confers immunomodulatory functions and remarkable tolerance against antimicrobials (Brennan and Nikaido, 1995) and organic solvents (Fernandes et al., 2003), enabling MCB to adapt to various niches.

Recent studies have reported that *Mycobacterium* species form MVs that have immunomodulating activity and iron-acquisition function (Prados-Rosales et al., 2011, 2014). For example, *Mycobacterium tuberculosis* and *Mycobacterium bovis* bacille Calmette-Guérin (BCG) have been shown to release MVs, which can induce an inflammatory response in mice lung in a TLR2-dependent manner (Prados-Rosales et al., 2011). Iron-deficient condition induced the release of siderophore-rich MVs in *M. tuberculosis*, suggesting a role of *M. tuberculosis* MVs in iron-acquisition for survival of the bacterium in host animal (Prados-Rosales et al., 2014). Although these observations have important implications on the biological significance of MVs in MCB, how MVs are triggered and formed in MCB remains a big question. Previous studies (Prados-Rosales et al., 2011, 2014) show *Mycobacterium* MVs form inner membrane vesicles (IMVs), further questioning the mechanisms of how they can traverse the cell wall and the mycomembrane. In addition, despite the presence of the mycomembrane in the MCB cells, MVs derived from the mycomembrane are scarcely reported and their formation mechanisms are largely unknown (Wang et al., 2020).

Here we show that the formation of two distinct types of MVs, namely, mycomembrane vesicle (mMV) and IMV, are induced in MCB due to different types of stress, such as DNA damaging stress, cell wall synthesis inhibition, or fatty acid biosynthesis inhibition (biotin depletion), which resemble the stress bacteria are exposed to in natural settings and have clinical significance (Carfrae et al., 2020; Hayashi et al., 2017; Kümmerer, 2003; Larsson, 2014; Park et al., 2011; Schaefer et al., 1955; Stallings and Glickman, 2010).

To understand how MCB form MVs in details, we examined MV formation in *Corynebacterium glutamicum* as a model organism*. C. glutamicum* is one of the most studied MCB and is a major player in the industrial production of valuable biomolecules such as glutamate. The cell envelope of *C. glutamicum* consists of an inner membrane, peptidoglycan, arabinogalactan, and a mycomembrane rich in corynomycolic acids (C32–C36 fatty acids; Figure 1A) (Bukovski, 2013; Hashimoto et al., 2006).

Using quick-freeze deep-etch electron microscopy (QFDE) and super resolution live cell imaging, we show that *C. glutamicum* MVs show different morphology and mode of formations depending on the inducing condition, and we further characterized the origin of each type of MVs by examining their lipid and protein compositions. Our results reveal that the complex envelope structure of *C. glutamicum* integrates the MV formation processes of both Gram-negative and Gram-positive bacteria, resulting in the generation of MVs with different origins through blebbing of the mycomembrane and bubbling cell death. These mechanisms were conserved in several MCB tested that have clinical relevance and may provide a platform for MV applications such as vaccine development. We also show that the localized site from which MVs are formed, such as cell poles, influence the MV composition, implying that the heterogeneous distribution of lipids and proteins in the membrane (Matsumoto et al., 2006; Toledo et al., 2018) may lead to generate heterogeneous MVs (Kikuchi et al., 2020). Although bacteria are thought as relatively simple single cell organisms and MVs formed from them are thought to be as simple, our finding provides basic knowledge on the different mechanisms of generating different types of MVs.

## Results

### DNA-damaging stress induces MV formation in *C. glutamicum*

Given the general role of DNA-damaging stress in MV formation of Gram-negative and Gram-positive bacteria ((Toyofuku et al., 2017a); Turnbull et al., 2016), we first investigated whether DNA-damaging stress would trigger MV formation in *C. glutamicum*. To evaluate the involvement of DNA damage, we treated *C. glutamicum* with mitomycin C (MMC), which is a genotoxic compound widely used in inducing DNA damage (Frunzke et al., 2008; Nanda et al., 2014). *C. glutamicum* ATCC13032 was grown to early exponential phase in the minimum medium (MM-1) as described in the Methods, and MMC was then added to the culture medium to a final concentration of 100 ng mL^−1^. After 12-h incubation, MVs were purified from the culture medium by density gradient ultracentrifugation, analyzed by nanoparticle tracking (Figure S1), and quantified using FM4-64, which stains the membrane. MV release increased in the MMC-treated cells compared with that of the control cells (Figures 1B, 1C, and S2).

MVs induced with MMC (M-MVs) were investigated using transmission electron microscopy (TEM) and were shown to be more diverse in morphology (spherical, tube-like, and shrunken) and in sizes than MVs formed under normal conditions (N-MVs) (Figure 1D). The ultrastructure of MVs was further investigated using QFDE that allows to observe the native structure of the samples at 1 nm resolution (Tulum et al., 2019). The spherical structures were also confirmed using QFDE and showed wrinkling of the surface and lipid bilayer structures (Figures 1E and S3). In addition, tube-like vesicle structures were occasionally observed in MVs induced under MMC conditions (M-MVs; Figures 1D and 1E).

DNA damage is shown to induce MV formation through the expression of peptidoglycan-degrading enzymes, endolysin, which are usually encoded in prophage regions of the genome (Andreoni et al., 2019)((Toyofuku et al., 2017a); Turnbull et al., 2016). *C. glutamicum* ATCC13032 possess three prophages (CGP1, CGP2, and CGP3) in its genome with a functional endolysin (NCgl1682) being encoded in CGP3 (Frunzke et al., 2008). When *NCgl1682* was deleted, M-MV formation was not triggered by MMC, but could be restored by *NCgl1682* complementation under control of its native promoter (Nanda et al., 2014) (Figure 1F). These results showed that MMC triggered MV formation through NCgl1682, further demonstrating the universal role of endolysin in MV formation.

### Endolysin triggered MV formation through cell death

**Figure 2:**
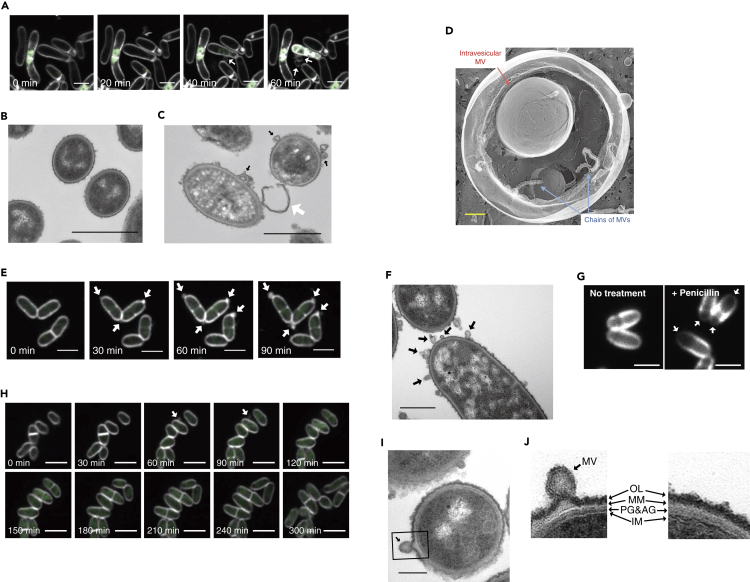
MV release by *Corynebacterium glutamicum* via different routes (A, E, and H) Live-cell imaging of MV formation of *C. glutamicum* under (A) MMC condition, (E) penicillin G, and (H) biotin-deficient conditions. The image shows FM4-64 (white) merged with SYTOX green (green). Movies are shown as Videos S1, S2, and S3. White arrows indicate MVs. Scale bars, 2 μm. (B and C) Thin-section TEM images of *C. glutamicum* cells under (B) no treatment and (C) MMC conditions. Black and white arrows indicate MVs and presumably a cell wall fragment, respectively. Scale bars, 1 μm. (D) Magnified QFDE image of MVs formed under penicillin G treatment condition. Red arrow and blue arrows indicate intravesicular MV and chains of MVs, respectively. Scale bar, 200 nm. (F) Thin-section TEM images of *C. glutamicum* cells under penicillin G. Black arrows indicate MVs. Scale bar, 1 μm. (G) Peptidoglycans of *C. glutamicum* cells were visualized using HADA in the presence or absence of penicillin G. White arrows indicate the cell pole in which peptidoglycan synthesis is severely inhibited by penicillin G treatment. Scale bars, 2 μm. (I) Thin-section TEM images of *C. glutamicum* cells under biotin-deficient conditions. Scale bar, 200 nm. (J) Magnified images of the cell envelope of an MV-forming cell (left, black square in I) and a non-forming cell (right). OL, outer layer; OM, outer membrane; PG, peptidoglycan; AG, arabinogalactan; IM, inner membrane.

Endolysin is known to trigger explosive cell lysis in Gram-negative bacteria or bubbling cell death in Gram-positive bacteria ((Toyofuku et al., 2017a); Turnbull et al., 2016). In explosive cell lysis, the cells “explode,” resulting in the shattered membrane to round up and form MVs, whereas in bubbling cell death MVs initially protrude from structurally intact cells. To understand the MV formation process in *C. glutamicum,* we performed super resolution live-cell imaging using confocal laser scanning microscopy (CLSM) with Airyscan detector. MV formation was observed from cells that were structurally intact (Figure 2A; Video S1). However, staining of cells with a membrane-impermeable dye (SYTOX green) indicated that the MV-forming cells had impaired membranes, similar to what has been observed in bubbling cell death of *B. subtili*s (Figure 2A; Video S1) (Toyofuku et al., 2017a).

Video S1. Time-lapse imaging of MMC-treated cells of *C. glutamicum*, related to Figure 2 The movie shows FM4-64 (white) merged with SYTOX green (green). Yellow arrow indicates MVs. Scale bar, 2 μm.

None of the MV-forming cells divided further during approximately 5 h of observation (Figure S4). This suggested that the cells died upon MV formation. Thin-section TEM and scanning electron microscopic (SEM) images confirmed that many MVs were associated with the surface of MMC-treated cells and were less electron dense in TEM compared with that of the untreated cells, suggesting cytoplasmic content had been released (Figures 2B, 2C, and S5).

### Cell envelope stress induced MV formation through blebbing of mycomembrane

Previous studies of Gram-negative bacteria have suggested that an imbalance of membrane and peptidoglycan synthesis eventually leads to outer membrane blebbing that pinches off to form MVs (Toyofuku et al., 2019)(Sutterlin et al., 2016). As MCB possess mycomembranes oustide the cell wall, we examined whether alterations in cell envelope synthesis would lead to MV release in *C. glutamicum*. To this end, we used penicillin G treatment and biotin-deficient conditions, which are reported to alter the cell envelope structure by inhibiting cell wall biosynthesis or fatty acid biosynthesis, receptively, in *C. glutamicum* (Hoischen and Kraman, 1990; Nakayama et al., 2018)*. C. glutamicum* was grown to early exponential phase in MM-1 medium, and then a sub-lethal concentration of penicillin G (0.4 U mL^−1^) was added to the culture. The penicillin G treatment of *C. glutamicum* induced MV release (Figures 1B–1D, S1, and S2). Interestingly, QFDE revealed that P-MVs (penicillin G-induced MVs) were usually internally packed with small MVs and chains of MVs, resulting in distinct structures compared with those of other types of MVs (Figure 2D). To determine the influence of biotin deficiency, which consequently decreases total amount of membrane lipids and alters membrane composition in the bacterium (Hoischen and Kraman, 1990; Nakayama et al., 2018), *C. glutamicum* cells were grown in biotin-deficient MM-1 medium (1 μg L^−1^ biotin). MV release was significantly increased under biotin-deficient conditions compared with that of biotin-sufficient conditions (100 μg L^−1^ biotin), indicating that biotin deficiency induced MV release in *C. glutamicum* (Figures 1B, 1C, S1, S2, and S5). Purified B-MVs (biotin deficiency-induced MVs) exhibited spherical and tube-like structures under TEM and QFDE, and tube-like MVs were observed more frequently than N-MVs and P-MVs (Figures 1D and 1E). It is well known that in *C. glutamicum* biotin deficiency and penicillin G treatment induce glutamate efflux through NCgl1221, a mechanosensitive channel (Nakamura et al., 2007; Nakayama et al., 2018; Nara et al., 1964; Shiio et al., 1962). The amount of glutamate or lysine contained in each purified MV fraction accounted for 0.015%–0.05% of the total glutamate or lysine content in the culture supernatant (Figures S6 and S7), indicating that glutamate and lysine were primarily released into the supernatant through NCgl1221, but not via MVs.

### Penicillin G induced MV formation in the cell pole of *C. glutamicum*

Different from MMC-treated cells, penicillin G-treated cells formed MVs from the cell pole (Figures 2E, S5, S8, and S9; Video S2). Cell permeability did not increase in the penicillin-treated cells (Figure 2E; Video S2) and approximately 10% of the MV-forming cells underwent cell division (n = 86; Figures S4 and S8). However, these cells stopped growing after MV release. Thin-section TEM of penicillin-treated cells showed MV blebbing from the cell surface (Figure 2F). As penicillin G inhibits cell wall synthesis, we visualized the effect of penicillin G on the cell wall of *C. glutamicum* using fluorescent labeling of the cell wall with HCC-amino-D-alanine (HADA), a synthetic D-amino acid with a fluorescent side chain (Kuru et al., 2012). The results showed that HADA fluorescence was barely detectable at the cell pole when the cells had been treated with penicillin G. However, HADA fluorescence was uniformly distributed along the cell envelope in the control cells not treated with penicillin G (Figure 2G and S10). These results indicated that penicillin G had more severe effect on the cell pole where cell wall would undergo frequent reorganization, and consequently inhibited cell growth. We assume that the inhibition of the peptidoglycan biosynthesis would make the mycomembrane-peptidoglycan linkage unstable, which would eventually lead to the blebbing of MVs.

Video S2. Time-lapse imaging of penicillin G-treated cells of *C. glutamicum*, related to Figure 2 The movie shows FM4-64 (white) merged with SYTOX green (green). Yellow arrows indicate MVs. Scale bar, 2 μm.

### Membrane stress by biotin deficiency induces MV formation in growing *C. glutamicum* cells

Under biotin-deficient conditions, over 80% of the MV-forming cells (n = 14) grew and divided after MV formation during the 5-h observation period of the study (Figure 2H and S4; Video S3). We believe this is the first clear evidence of cell division after MV release, supporting a canonical MV blebbing model of MVs being formed during cell growth (Beveridge, 1999). MV formation by *C. glutamicum* under biotin-sufficient conditions was not observed using CLSM due to lower MV formation and faster cell growth compared with that under biotin-deficient conditions. Interestingly, thin-section TEM of cells under biotin-deficient condition revealed protrusions of the cell envelope where B-MVs were observed (Figures 2I and 2J). These protrusions were observed with significantly higher frequency at the sites of MV formation than in the sites where no MV formation was observed (Figure S11), whereas the protrusions were seldomly observed on cells grown under normal conditions (Figure 2B). These results suggest that an imbalance in the synthesis of the cell envelope or accumulation of substances such as proteins may have led to the membrane protrusions that formed the MVs.

Video S3. Time-lapse imaging of *C. glutamicum* cells in biotin deficiency, related to Figure 2 The movie shows FM4-64 (white) merged with SYTOX green (green). Yellow arrow indicates MV. Scale bar, 2 μm.

### MV lipid composition indicates different origins of MVs

**Figure 3:**
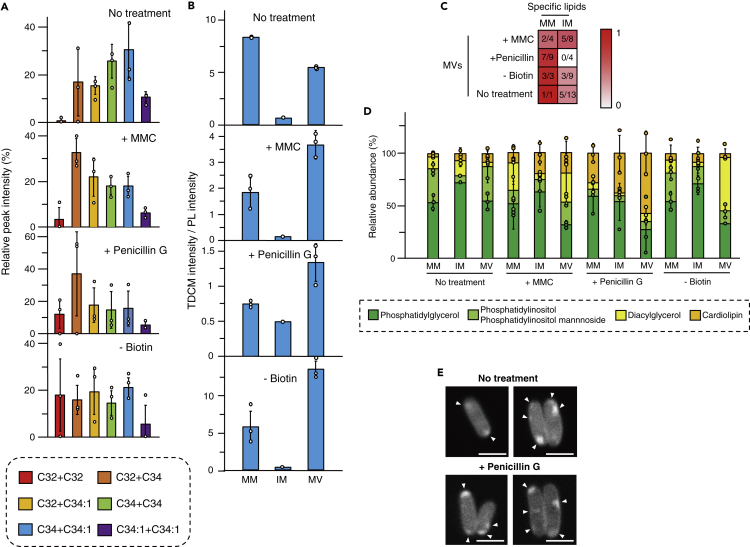
Lipid compositions of MVs (A) Trehalose dicorynomycolic acids (TDCMs) were detected from MVs using LC/MS. The structure of each TDCM was determined based on the results of GC/MS and LC/MS/MS analyses shown in Figures S12 and S14. (B) Intensities of TDCM and phospholipids (PLs) in TLC analysis were compared using ImageJ. Each lipid was extracted from mycomembrane (MM), inner membrane (IM), and MV of *Corynebacterium glutamicum*, and then separated by TLC as described under Methods. All values indicated by the bars represent the mean value ± SD for three experiments. (C) Mycomembrane-specific lipids (MMSLs) and inner membrane-specific lipids (IMSLs) of *C. glutamicum* cells under various growth conditions were identified by LC/MS analyses. In these analyses, we defined MMSLs and IMSLs as the lipids that were detected in either the mycomembrane (1-butanol extract) or the inner membrane extract (total of chloroform/methanol and chloroform/methanol/water extracts) of *C. glutamicum* cell under each of the designated culture conditions. Detailed information of these analyses is shown in Methods and Figures S15–S18. Denominator of each fraction indicates total number of specific lipids in the mycomembrane or the inner membrane extract of *C. glutamicum* cells under different culture condition, and the numerator of each fraction indicates total number of those MMSLs or IMSLs detected in MVs. (D) Lipid compositions of mycomembrane, inner membrane, and MVs were analyzed. Mycomembranes and inner membranes were extracted separately from cells using 1-butanol and chloroform/methanol solutions, respectively. Lipids were quantified using TLC and standard lipids. All values indicated by the bars represent the mean value ± SD for three experiments. (E) Cardiolipins were visualized using acridine orange 10-nonyl bromide. White arrowheads indicate the localization of cardiolipins. Scale bars, 2 μm.

Live-cell imaging indicated that *C. glutamicum* was able to form MVs through different process under different conditions. This led to our hypothesis that MVs may differ in their composition. Therefore, MV lipid content of different types of MVs, including MVs isolated from culture without any treatment (N-MVs), was analyzed using gas chromatography/mass spectrometry (GC/MS) and liquid chromatography/mass spectrometry (LC/MS). Corynomycolic acids (CMs) (Bukovski, 2013; Hashimoto et al., 2006), which are major components of *C. glutamicum* mycomembrane (Figure 1A), were detected in all types of MVs (Figures 3A and S12). This provided a general feature of MVs released from this bacterium. Most of the CMs detected were trehalose dicorynomycolic acids (TDCMs), which are found in the outer leaflet of the CM bilayer and are not covalently bound to the cells' arabinogalactan (Figures S12–S14) (Bukovski, 2013). To gain more insight into the origins of *C. glutamicum* MVs, we extracted mycomembrane and inner membrane lipids separately from *C. glutamicum* cell (described under Methods). The selectivity of the membrane lipid separation is supported by the observation that the mycomembrane extracts were apparently colorless, whereas the inner membrane extracts were yellow-pigmented, indicating the presence of renoxanthin (a C_50_ carotenoid), which localizes in the inner membrane (Sandmann ans Yukawa, 2005). We then separated and detected the major lipids from each membrane and MV fractions by thin-layer chromatography (TLC; Figure S15) and compared the intensity ratios of TDCMs/phospholipids (Figure 3B). Overall, the intensity ratios of TDCMs/phospholipids were higher in MVs than in the inner membrane fraction, suggesting that the MVs contained lipid components of the mycomembrane. However, a comprehensive analysis and comparison of the lipid composition among the mycomembrane, inner membrane, and MVs (Figures S16–S18; described under Methods) showed that the N-MVs fraction, P-MVs fraction, and B-MVs fraction had more similar composition to the mycomembrane than that to the inner membrane fraction, whereas M-MVs fraction had lipid composition more similar to that of the inner membrane fraction (Figures 3C and S16–S18). Most remarkably, P-MVs fraction contained most of the mycomembrane-specific lipids (7 of 9) but none of the inner membrane-specific lipids (Figures 3C and S16–S18). The presence of inner membrane lipids in N-MVs and B-MVs fractions may be due to the formation of IMVs resulting from the basal level of cell death in cell cultures, whereas presence of IMVs was overcovered by mMVs in case of P-MVs. Notably, the lipid profile of N-MVs fraction was different from those of M-MVs, P-MVs, and B-MVs fractions (Figures S16–S18), and the addition of biotin to the culture failed to decrease N-MV release (Figure S19), indicating that N-MVs were formed through a mechanism different than that of B-MVs. The drastic changes in the cellular membrane lipid compositions among the growth conditions, especially MMC and penicillin G conditions, suggest stress-responsive membrane remodeling caused by MalR (Hünnefeld et al., 2019), a MarR-type regulator that has been proposed to be involved in adaptation of *C. glutamicum* cells to DNA stress and inhibition of cell wall biosynthesis. The previous study (Hünnefeld et al., 2019) has identified binding sites of MalR in *C. glutamicum* genomes and revealed that MalR regulates expressions of several genes, such as *ipsA* and *oppA*, involved in cell membrane remodeling. Positive and negative regulation of these genes by MalR may result in modulation of lipid composition of *C. glutamicum* membranes upon the cell envelope and DNA stresses.

To determine whether the presence of mycomembrane was essential for the induction of MV formation, we constructed a Δ*pks13* mutant of *C. glutamicum*, which lacked corynomycolic acids and a mycomembrane (Portevin et al., 2004; Zuber et al., 2008). This mutant exhibited less MV release than that of the wild-type strain under normal growth conditions (Figure S20). Moreover, MV release tends to be induced with MMC treatment of the mutant, but not with biotin deficiency or penicillin G treatment (Figure S20). This further indicates that outer membrane is essential for the induction of MV release in *C. glutamicum* under the latter two envelope stress conditions, whereas IMVs formation can be trigged by degradation of the cell wall in the mutant cells.

Comparison of the lipid groups showed that the ratios of phosphatidylglycerol (PG), phosphatidylinositol, diacylglycerol (DG), and cardiolipin (CL) differed depending on the MVs (Figures 3D and S15). The conical shape of DG is reported to form a negative curvature in membranes, which supports budding in eukaryotes (Agrawal and Ramachandran, 2019). We found that DG was enriched in B-MVs fraction, implying a similar role in MV formation under biotin-deficient conditions. CL is another conical-shaped lipid and was found to be enriched in P-MVs fraction (Figures 3D and S15). CL consists of two PGs that are linked by a glycerol head group and are major membrane components in bacteria and mitochondria (Mileykovskaya and Dowhan, 2009). CL has been found to accumulate at the cell pole (Mileykovskaya and Dowhan, 2000) and at curved regions of membranes (Renner and Weibel, 2011) of bacteria, suggesting that the clustering of CL may cause membrane curvature in bacterial membranes, and may also lead to the formation of MV chains. Cell imaging showed that CL tended to localize at the cell pole of *C. glutamicum* (Figure 3E), consistent with our results of penicillin G treatment leading to MV formation in the cell pole (Figures 2E and 2F). In addition, CL content was high in the cell membrane of penicillin G-treated cells (Figure 3D), suggesting that the treatment led to remodeling of the membrane composition. Given that expression of CL synthase (NCgl2646) has been reported to be elevated upon penicillin G treatment in *C. glutamicum* (Hirasawa et al., 2018), the higher CL content in the penicillin G-treated cells may be due to increased *de novo* biosynthesis of CL. In addition, cell wall-associated CL, which is not extractable from the cells under normal growth condition (Bansal-Mutalik and Nikaido, 2011), may be released upon cell wall damaging by penicillin G and localize in cell membrane as extractable lipid.

### M-MVs, but not other MVs, included cytoplasmic substances

**Figure 4:**
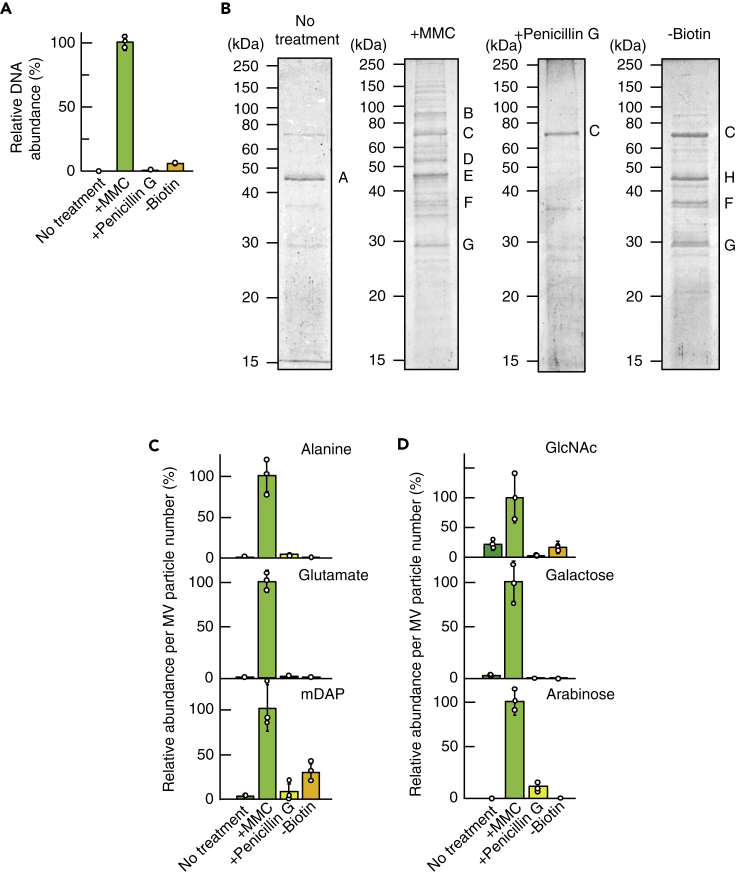
Detection of various cellular components in MVs (A) Quantification of concentrations of double-stranded DNA associated with *C. glutamicum* MVs. All values indicated by the bars represent the mean value ± SD for three experiments. (B) Protein profiles of MVs. A, hypothetical membrane protein (NCgl0381); B, 5-methyltetrahydropteroyltriglutamate-homocysteine *S*-methyltransferase; C, PS1; D, ATP synthase β-subunit; E, elongation factor Tu; F, corynomycoloyl transferase C chain A (Cmt1); G, esterase family protein (Cmt2); H, PS1 fragment. Five micrograms of protein was applied to each lane. (C and D) Detection and quantification of (C) amino acids and (D) sugars that are derived from cell wall fragments in MVs. All values indicated by the bars represent the mean value ± SD for three experiments.

Many MVs have been shown to carry cytoplasmic materials such as DNA as cargo, and it has been proposed that MVs blebbing from the outer membrane do not contain cytoplasmic material, whereas MVs generated through cell lysis or cell death contain cytoplasmic material in their cargo (Toyofuku et al., 2019). To better understand the origins of MVs, we quantified the amount of double-stranded DNA (dsDNA) in the MVs. The results clearly demonstrated that dsDNA was most abundant in the M-MVs and was scarcely detected in the other types of MVs (Figure 4A). In addition, we conducted mass spectrometric analysis to identify proteins enriched in the MVs. Proteins in MVs were separated by gel electrophoresis, and the major proteins were subjected to mass spectrometry (details are described under Methods). Consistent with the result of DNA quantification, cytosolic proteins 5-methyltetrahydropteroyltriglutamate-homocysteine *S*-methyltransferase and elongation factor Tu and inner membrane protein ATP synthase β-subunit were abundant in M-MVs, but not detected in the other MVs (Figure 4B). Surprisingly few protein bands were detected by SDS-PAGE analysis of N-MVs and P-MVs (Figures 4B and S21). NCgl0381 is a hypothetical membrane protein containing a predicted N-terminal secretion signal peptide sequence and was predominantly detected in N-MVs (Figures 4B and S22). The most abundant protein in P-MVs was PS1, a mycoloyl transferase that is involved in the biosynthesis of TDCMs (Puech et al., 2000). PS1 is associated with the cell envelope and also secreted into the culture medium (Brand et al., 2003). Furthermore, corynomycoloyl transferase C chain A (Cmt1) and esterase family protein (Cmt2), which are involved in the biosynthesis of TDCM and localize in the cell envelope (Brand et al., 2003), were detected in B-MVs (Figure 4B). CspB, which was previously reported to be abundant in MVs induced by EDTA treatment of *C. glutamicum* ATCC13869 (Theresia et al., 2018), was not detected in MVs analyzed in the current study due to the absence of this protein in the strain we used (Yang and Yang, 2017). These results support the idea that N-MVs, P-MVs, and B-MVs were primarily formed through mycomembrane blebbing, whereas M-MVs were formed through bubbling cell death, which leads to inner membrane protrusion and liberation of the mycomembrane due to cell wall degradation.

### Cell wall components were associated with M-MVs

Cell wall components, such as peptidoglycan, are typical MV cargo that have important roles in host immune modulation including *M. tuberculosis* (Kaparakis-Liaskos and Ferrero, 2015; Prados-Rosales et al., 2011). However, the process of how the cell wall components are packaged into MVs is not fully understood. In *C. glutamicum,* the major peptide units in the cell wall are L-Ala-D-Glu-*meso*-diamiopimelate (mDAP)-D-Ala and L-Ala-D-Glu-mDAP, which are peptide units cross-linked via mDAP-mDAP bridges (Bukovski, 2013). In addition to these amino acids, *N*-acetylglucosamine (a component of the peptidoglycan backbone) and arabinose and galactose (components of arabinogalactan) are contained in the cell wall (Bukovski, 2013). These molecules were most abundant in M-MVs and scarcely detected in the other types of MVs, further suggesting that cell wall components were packaged into MVs in *C. glutamicum* by degradation of the cell wall (Figures 4C, 4D, and S23). In addition, the molar ratios of Ala/Glu/mDAP were approximately 1.4/1/0.015 in M-MVs compared with approximately 1.4/1/1 in the cells (Figure S24). This suggested that mDAP-mDAP bridges in the peptide units were hydrolyzed by endolysin.

### Induction of MV formation among mycolic acid-containing bacteria

**Figure 5:**
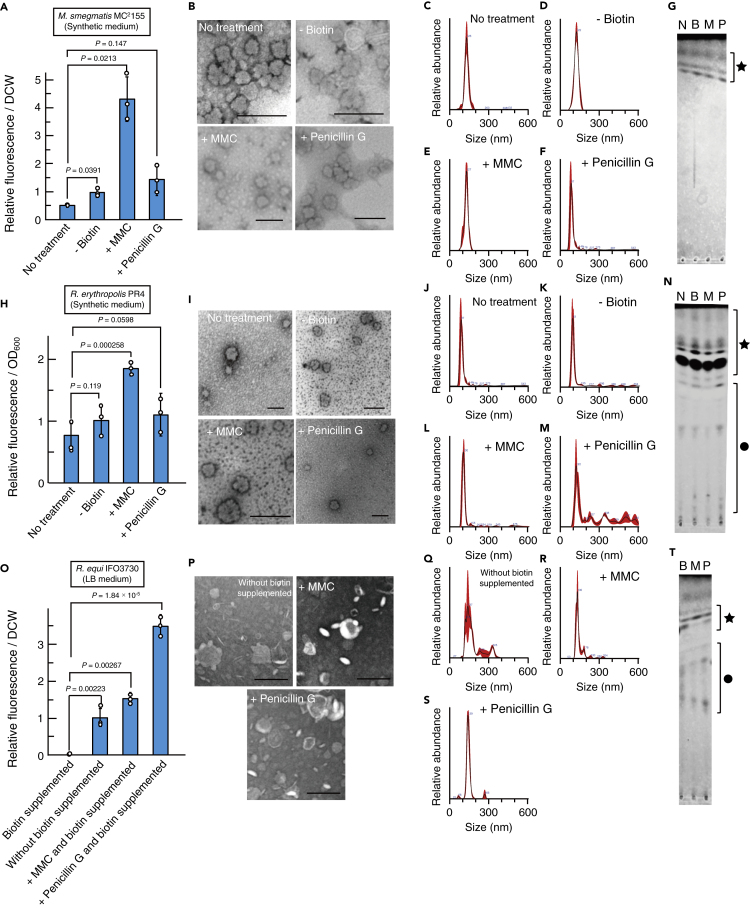
MV induction in other mycolic acid-containing bacteria (A–T) The panels (A–G, H–N, and O–T) correspond to *Mycobacterium smegmatis* MC^2^155, *R. erythropolis* PR4, and *Rhodococcus equi* IFO3730, respectively. (A, H, and O) *M. smegmatis*, *R. erythropolis*, and *R. equi* were cultured under various conditions and then their MV release were measured. *M. smegmatis* and *R. erythropolis* were cultured in synthetic minimum media, whereas *R. equi* was cultured in LB medium, with and without biotin supplementation, due to extremely low growth in synthetic minimum medium (details of growth conditions are described under Methods). All values indicated by the bars represent the mean value ± SD for three experiments. p values were calculated using unpaired t test with Welch's correction. DCW, dried cell weights. (B, I, and P) TEM images of MVs of the mycolic acid containing bacteria are shown. Scale bars, 200 nm. (C–F, J–M, and Q–S) Particle size distributions of the above MVs. Black lines indicate the mean values of the concentrations of the detected particles in MV solutions. Red regions indicate SD of the mean values. (G, N, and T) Thin-layer chromatography profiles of the above MVs. Lipids were processed using chloroform-methanol-water (65:25:4, v/v). N, no treatment condition; B, biotin-deficient condition in *M. smegmatis* and *R. erythropolis*, or without biotin supplemented in *R. equi*; M, MMC treatment condition; P, penicillin G treatment condition. Black star indicates apolar lipids including mycolic acid esters. Black circle indicates polar lipids including phospholipids.

As MCB share cell envelope structural features (Figure 1A), we investigated whether MV formation was also induced in *Mycobacterium* and *Rhodococcus* species by biotin deficiency, MMC treatment, and/or penicillin G treatment. We found that MV release was induced in *Mycobacterium smegmatis* MC^2^155 (Figures 5A–5G), *Rhodococcus erythropolis* PR4 (NBRC100887) (Figures 5H–5N), and *Rhodococcus equi* IFO3730 (Figures 5O and 5T) under the aforementioned conditions. This is also supported by bioinformatic analyses indicating the presence of lytic enzymes in prophages regions of MCB. Genomic search by PHASTER (Arndt et al., 2016) showed that *M. smegmatis* MC^2^155 (putative alpha/beta hydrolases, LJ00_07,870 and LJ00_07,930) and *R. erythropolis* PR4 (putative endolysin, RER_22,520) possess prophages encoding putative lytic enzymes in their genome. Although whole genome information of *R. equi* IFO3730 is not available, PHASTER search revealed that the related strain, *R. equi* ATCC33707, also possesses two prophages in its genome and at least one putative endolysin (HMPREF0724_RS24760) is coded in the region. Of note, we cultured *R. equi* in LB medium supplemented with 200 μg L^−1^ biotin as MVs in minimum medium was too low to quantify. MV release by *R. equi* significantly increased, even when LB medium was not supplemented with biotin as this medium already contained approximately 1.5 μg L^−1^ biotin (Figure 5O) (Difco Laboratories, 1998). Size distributions of MCB MVs (Figures 5C–5F, 5J–5M, and 5Q–5S) suggest that the diameter ranges of most of these MVs (approximately 80–200 nm) are similar to those of typical bacterial MVs including previously reported *M. smegmatis* MVs (Prados-Rosales et al., 2011).

## Discussion

MV formation has been widely studied in Gram-negative and Gram-positive bacteria, but little is known about MV formation in MCB, which are grouped as Gram-positive bacteria possessing a mycomembrane in addition to a thick cell wall. Previous studies in *Mycobacterium* species have suggested that they mainly form IMVs (Prados-Rosales et al., 2011, 2014), and are induced under iron starvation, which also alters their composition (Prados-Rosales et al., 2014), raising the following fundamental questions: (1) how are IMVs formed and released through the cell wall and the mycomembrane, (2) what causes the change of MV composition under different culture conditions, and (3) how are these MVs induced. Our results show that formation and release of different types of MVs by MCB can be induced through different routes involving the mycomembrane blebbing and bubbling cell death depending on different conditions. Membrane stress or cell wall synthesis inhibition, which presumably leads to the loss of linkage between the cell wall and the mycomembrane, induces mycomembrane blebbing, forming mainly mMVs. Cell wall disruption led to bubbling cell death forming a mixture of mMVs and IMVs. The involvement of cell lysis in MV formation would be one of the mechanisms for IMVs release, and importantly, cytoplasmic and cell wall components were associated with MVs triggered by bubbling cell death.

Distinct routes for MV formation have also been reported in other groups of bacteria. In Gram-negative bacteria, MVs can be formed through blebbing of the outer membrane (Schwechheimer and Kuehn, 2015) or explosive cell lysis (Toyofuku et al., 2019; Turnbull et al., 2016). Bona fide OMVs formed through outer membrane blebbing without cell lysis are suggested to contain limited intracellular components and inner membrane proteins (Toyofuku et al., 2019; Schwechheimer and Kuehn, 2015), whereas MVs formed through explosive cell lysis may contain inner membrane and cytoplasmic materials (Turnbull et al., 2016). In Gram-positive bacteria, MV are formed by alteration or damaging of the cell wall through which the cytoplasmic membrane protrudes and MVs are pinched off (Andreoni et al., 2019)((Toyofuku et al., 2017a); Wang et al., 2018). MV-like particles were observed on the surface of *C. glutamicum* cells under conditions of biotin deficiency (Ochiai et al., 1987) or dysfunction in cell wall biosynthesis (Raad et al., 2010), although they were not characterized at that time. Using super resolution live-cell imaging and biochemical analysis, we showed that lysing *C. glutamicum* cells release IMVs and mMVs through bubbling cell death under MMC treatment condition (Figure 1), and the cells under biotin-deficient condition or penicillin G treatment condition release mMVs through the mycomembrane blebbing without cell lysis (Figure 1). It has been long proposed that OMVs are formed only from growing cells,even though direct evidence using live-cell imaging was lacking (Schwechheimer and Kuehn, 2015). We show that both growing biotin-deficient *C. glutamicum* cells and penicillin G-treated cells release MVs whose compositions indicate their mycomembrane origin, suggesting that mycomembrane blebbing can happen in both dead and alive cells depending on the mechanisms. The involvement of endolysin in MV formation by *C. glutamicum* provides further evidence of its universal role in MV formation among structurally distinct bacteria ((Toyofuku et al., 2017a); Turnbull et al., 2016) and could be a major route for MV formation in natural environments considering their abundance and the numerous mycobacteriophages isolated (Hatfull, 2018). Although the involvement of bubbling cell death resembles an earlier observation in MV formation in *B. subtilis* (Toyofuku et al., 2017a), it is distinct in that bubbling cell death in *C. glutamicum* also gives rise to mMVs. The MV formation mechanisms characterized in *C. glutamicum* were suggested to be conserved in other MCB tested, including *M. smegmatis*, which has been used as a non-pathogenetic model organism of *M. tuberculosis*, one of the most productive killers among infectious diseases (Smith, 2003). Our results show that due to the rigid cell wall structures and the presence of a mycomembrane in MCB, MV formation in these bacteria reflects the features of both Gram-negative and Gram-positive bacteria, resulting in various MVs being formed with different lipid and protein contents.

Previous studies have shown that lipid and protein profiles often differ between MVs and cellular membrane, suggesting cargo selection during MV biogenesis, whose mechanism is not fully understood (Nagakubo et al., 2020; ; Schwechheimer and Kuehn, 2015). Our study shows that P-MVs formed at the cell pole are enriched in CL and have a different protein profile compared with N-MVs, strongly suggesting that the subcellular localizations of MV formations can generate different type of MVs due to the heterogeneous distribution of lipids and proteins in the membrane. In addition, the accumulation of CL in P-MVs and the cellular membranes under penicillin G condition may also imply its role in MV formation mechanism. Besides CL, B-MVs released under biotin-deficient condition accumulated DG, a minor component of *C. glutamicum* membrane. Notably, CL and DG represent bacterial cone-shaped lipids whose accumulation or sequestration potentially cause membrane curvature (Agrawal and Ramachandran, 2019) and possibly the resultant pinching-off of MVs. It is thus possible that the remodeling of membrane lipid compositions and the enrichment of the cone-shaped lipids in response to the specific growth conditions triggers the membrane budding and the consequent MV formation with the distinct lipid compositions. This is supported by the previous study showing that *Haemophilus influenzae* released OMVs rich in phosphatidylethanolamine (PE), another typical cone-shaped lipid in bacterial membranes, under iron limitation condition (Roier et al., 2016). Together with our results, this and the related study in *Vibrio cholerae* (Zingl et al., 2019) suggest that the remodeling membrane lipid compositions and the accumulation of lipids with certain conformational properties may be a key to understanding a common molecular mechanism underlying MV formation across diverse classes of bacteria.

P-MVs showed a characteristic structure of MVs being packed with smaller MVs, creating multivesicular MVs. How the various MV compositions impact MV morphology is of interest, but currently largely unexplored. Multivesicular structures are observed in biological systems such as multivesicular endosomes, and although the process is not fully understood, the presence of certain lipids and protein complexes are known to play a role in their formation (Matsuo et al., 2004; Trajkovic et al., 2008; Wollert and Hurley, 2010). The unique MV structure of P-MVs may due to the enrichment of CL from the cell pole causing a curvature of the membrane (Agrawal and Ramachandran, 2019; Mileykovskaya and Dowhan, 2000, 2009), rather than a resultant of sample preparation.

In addition, the proteins detected in the current study, PS1 and NCgl0381, which were dominant in P-MVs and N-MVs, respectively (Figure 4B), may potentially be used as a basis for selective compartmentalization of certain proteins using protein fusion to generate specific MVs for various applications in biotechnology, such as purification of heterologously expressed proteins and vaccine development.

Our finding of the involvement of biotin in MV formation has a broad implication. Biotin is an essential biological cofactor involved in key metabolic pathways, including fatty acid biosynthesis, suggesting biotin limitation as a universal factor in bacterial MV formation. Some bacteria are auxotrophic for biotin, including *M. tuberculosis* clinical isolates (Schaefer et al., 1955), and many bacteria that can synthesize biotin are known to possess transporters to uptake biotin from the environment (Hebbeln et al., 2007), implying that biotin limitation may occur among these bacteria. Furthermore, mammals rely on diet and gut microbes for biotin supply, and it is tempting to speculate that biotin has important roles in host-microbe interaction (Hebbeln et al., 2007; Yoshii et al., 2019), where MVs may also take part. Biotin synthesis has been reported to be essential for *M. tuberculosis* acute infection (Park et al., 2011; Sassetti and Rubin, 2003), and as mammals lack biotin biosynthetic enzymes, biotin biosynthesis is a potential target for antibiotic development (Park et al., 2011), with amiclenomycin and actithiazic acid being examples of such antibiotics that work especially well against mycobacteria (Salaemae et al., 2016). Penicillin G is also reported to be effective for infectious diseases caused by some MCB pathogens including *R. equi* in combination with other antibiotics (Jacks et al., 2003), and our results suggest that antibiotics targeting biotin biosynthesis and cell wall biosynthesis may enhance MV formation, similar to what is observed in other bacteria (Andreoni et al., 2019)(Fulsundar et al., 2014; Manning and Kuehn, 2011).

The involvement of antibiotics in MV formation also has ecological significance as many antibiotics that inhibit DNA replication or the biosynthesis of bacterial cell wall have been found to be produced by bacteria and fungi (Levine, 2006; Umezawa et al., 1966). For example, MMC and penicillin G, which induced MV formation, were originally isolated from *Streptomyces caespitosus* (bacterium) or *Penicillium chrysogenum* (fungus), respectively (Gaynes, 2017; Wakaki et al., 1958). These antibiotic-producing microorganisms are known to exist in complex ecological systems such as soil in which MCB have also been found (Huska and Kaevska, 2012; Komukai-Nakamura et al., 1996; Takai et al., 1991; Udaka, 1960) and may coexist in such environments. Thus, it is possible that MCB experience DNA stress and cell-envelope stress in the environment that generate different types of MVs giving various roles in cell-to-cell interactions (Brown et al., 2015; Domingues and Nielsen, 2017; Kadurugamuwa and Beveridge, 1996; Mashburn and Whitley, 2005; , 2017b; Schwechheimer and Kuehn, 2015).

Our findings would have clinical relevance as some strains of *R. equi* are zoonotic and cause severe pyogranulomatous pneumonia in young horses and immunocompromised humans (Hondalus, 1997). Moreover, *M. smegmatis* is widely used as a non-pathogenic model organism of *M. tuberculosis* and have similar cell envelope structures (Alderwick et al., 2015). Host-induced stresses may ultimately induce MV formation in MCB pathogens, as these pathogens experience DNA damage and attacks of antimicrobial peptides and proteins that cause membrane destabilization during infection (Stallings and Glickman, 2010). A recent study reported that MVs released from *M. avium,* under conditions mimicking the macrophage phagosome, contain dsDNA (Chiplunkar et al., 2019) Notably, it was shown that IMVs released from *M. tuberculosis* are involved in their pathogenesis in mice by modulating immune responses in a TLR2-dependent manner (Prados-Rosales et al., 2011). These observations suggest that IMVs play important roles in the pathogenesis of pathogenic MCB. Our results further show that IMVs are only formed through endolysin-triggered bubbling cell death, and dsDNA is abundant in MVs triggered by bubbling cell death, suggesting that cell wall degradation or alterations are involved in the biogenesis of the MVs observed in the earlier studies. Future work on elucidating each role of different types of MVs may provide us an overall understanding of how MVs are involved in the pathogenicity of MCBs. Finally, as MVs have drawn great attention in application due to its potential as serving as a platform for vaccine development (Acevedo et al., 2014), the basic knowledges in inducing MV formation may facilitate vaccine development against pathogenic MCB based on various types of MVs that are released by the pathogens.

### Limitations of study

Although we characterized different MV formation routes in *C. glutamicum*, the biological function of *C. glutamicum* MVs are currently unknown. Accumulating evidences indicate that MVs play roles in immunomodulation and nutrient acquisition in other MCB, but the knowledge about the MV function in these bacteria are limited. Our next challenge would be to unravel the biological function of *C. glutamicum* MVs and how the difference in the formation routes impacts their functions.

In the biochemical analyses for MV composition, we focus on the abundance of the major proteins, which are apparently enriched in certain types of MVs, to clarify the origins of *C. glutamicum* MVs released under different conditions. The abundance of the minor proteins is not described here, and a more intensive proteomics approach may hint the function of the MVs. In addition, there is a potential limitation in our lipid analysis. Although comprehensive lipid analysis clarified mycomembrane- and inner membrane-specific lipids in *C. glutamicum,* which can be used as markers for determining membrane origins of MVs, the number of these membrane-specific lipids might be underestimated due to limitation in membrane separation selectivity. Finally, we could not determine whether N-MVs formed under normal growing conditions are released from viable cells or dying cells due to fast cell growth and low frequency of MV formation in *C. glutamicum* cells under the tested condition. Considering the biochemical compositions of N-MVs indicating their mycomembrane origin, they are presumably released from viable cells, but this requires further investigation.

### Resource availability

#### Lead contact

Further information and requests for resources and reagents should be directed to and will be fulfilled by the Lead Contact, Masanori Toyofuku (toyofuku.masanori.gf@u.tsukuba.ac.jp).

#### Materials availability

Plasmids and bacterial strains generated in this study are available from the Lead Contact with a completed Materials Transfer Agreement.

#### Data and code availability

The datasets supporting the current study are available from the corresponding author on request.

## Methods

All methods can be found in the accompanying Transparent Methods supplemental file.

## Supplemental information

Document S1. Transparent methods, Figures S1–S24, and Tables S1 and S2
