# Supplementary material for: Mycolic acid-containing bacteria trigger distinct types of membrane vesicles through different routes
Source: iScience. 2021 Jan 14;24(1):102015. doi: 10.1016/j.isci.2020.102015 (PMC7835258; doi:10.1016/j.isci.2020.102015)
Supplement: Document S1. Transparent methods, Figures S1–S24, and Tables S1 and S2 [file mmc1.pdf]

iScience, Volume *24*

Supplemental Information

Mycolic acid-containing bacteria trigger distinct types of membrane vesicles through different routes

Toshiki Nagakubo, Yuhei O. Tahara, Makoto Miyata, Nobuhiko Nomura, and Masanori Toyofuku

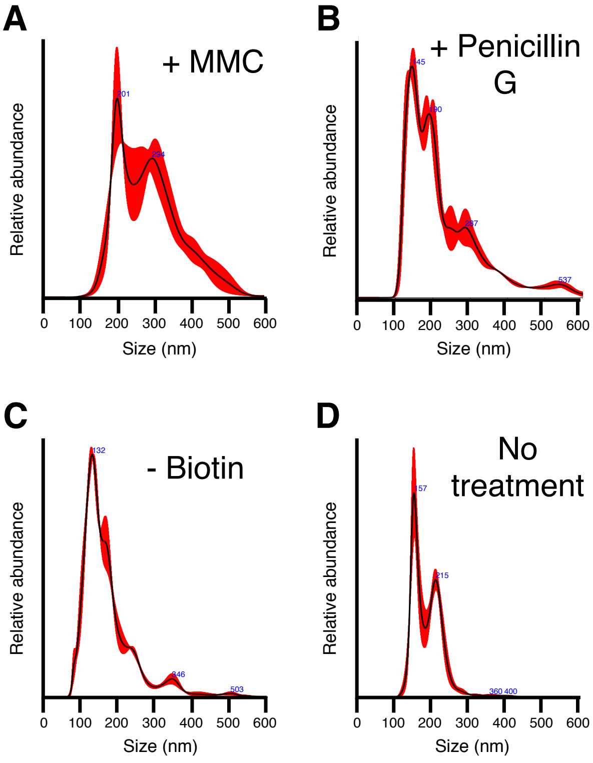

Supplemental Figure 1. Size distributions of MVs. Related to Fig. 1.

Particle size distributions of (A) M-MVs, (B) P-MVs, (C) B-MVs and (D) N-MVs. Black lines indicate the mean values of the relative concentrations of the detected particles for three experiments. Red regions indicate ± S. D. of the mean values.

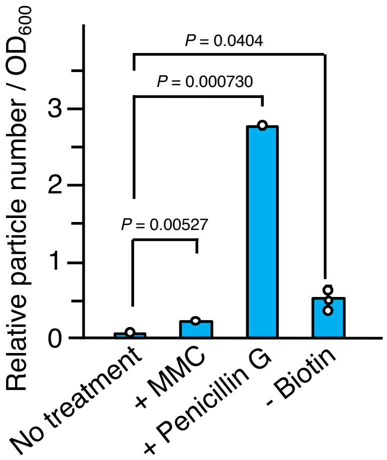

Supplemental Figure 2. Relative concentrations of *Corynebacterium glutamicum* MVs. Related to Fig. 1.

C. glutamicum MVs were analysed by nanoparticle tracking using Nanosight and the calculated particle concentrations were normalised to OD_{600}. *P* values were calculated using unpaired *t*-test with Welch's correction. All values indicated by the bars are the mean ± S. D. for three experiments.

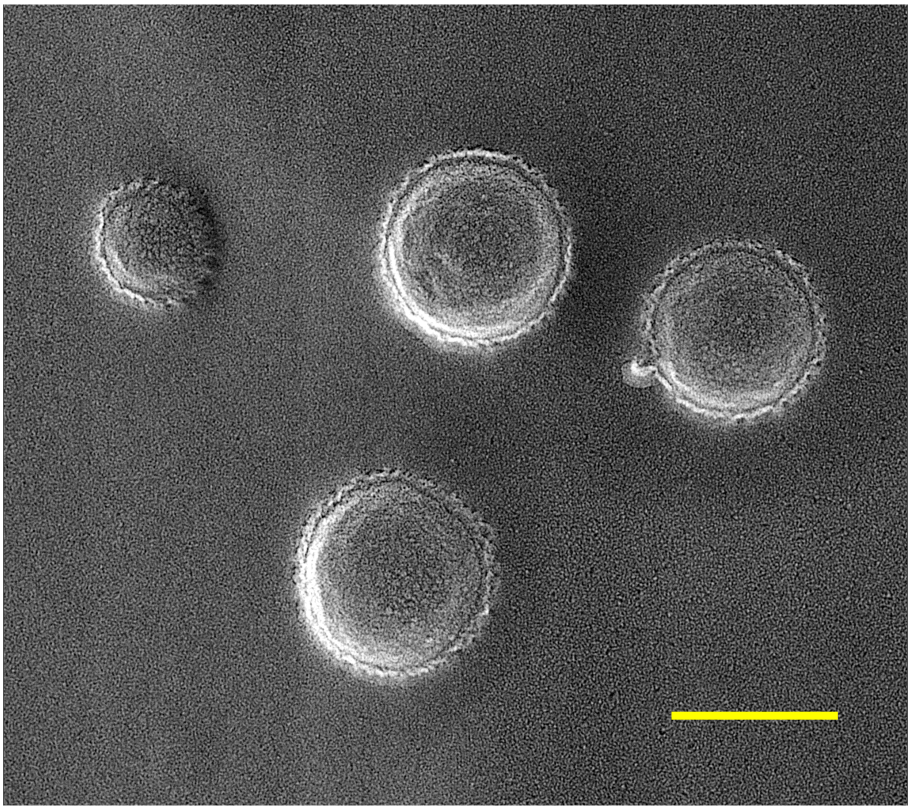

Supplemental Figure 3. QFDE image of N-MVs. Related to Fig. 1.

QFDE image of N-MVs shows lipid bilayer structures. Scale bar, 200 nm. The image shows MVs whose outer leaflet of the lipid bilayer was stripped off, exposing the inner leaflet of the lipid bilayer. The outer leaflet of the lipid bilayer was stripped off due to the tendency of the fracture plane to follow a plane through the hydrophobic core of the membrane at cryogenic temperature. Glycerol was added before cryo fixation.

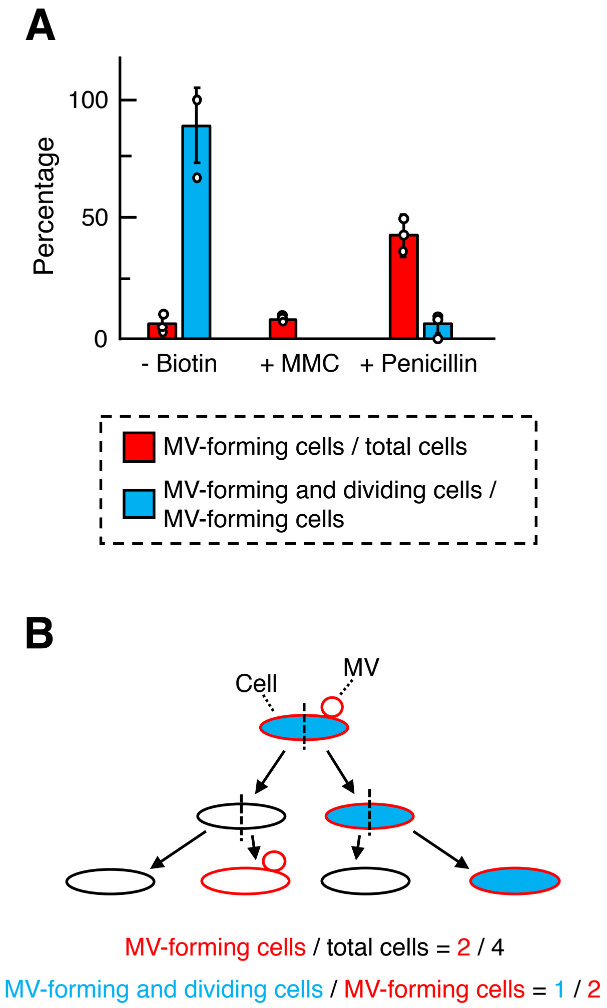

Supplemental Figure 4. Populations of MV-forming cells. Related to Fig. 2.

Populations of MV-forming cells were counted during time-lapse imaging using CLSM. (A) Percentages of MV-forming cells per total cells (red bars) and MV-forming and growing cells per total MV-forming cells (blue bars) were calculated. Cells that divided during the observation for ~5 hours were defined as dividing cells. All values indicated by the bars represent the mean value ± S.D. for 3 fields. In total, 208, 413, and 206 cells were counted in biotin deficiency, under MMC and penicillin G conditions, respectively. (B) Schematic diagram of a method for counting MV-forming cells in this study.

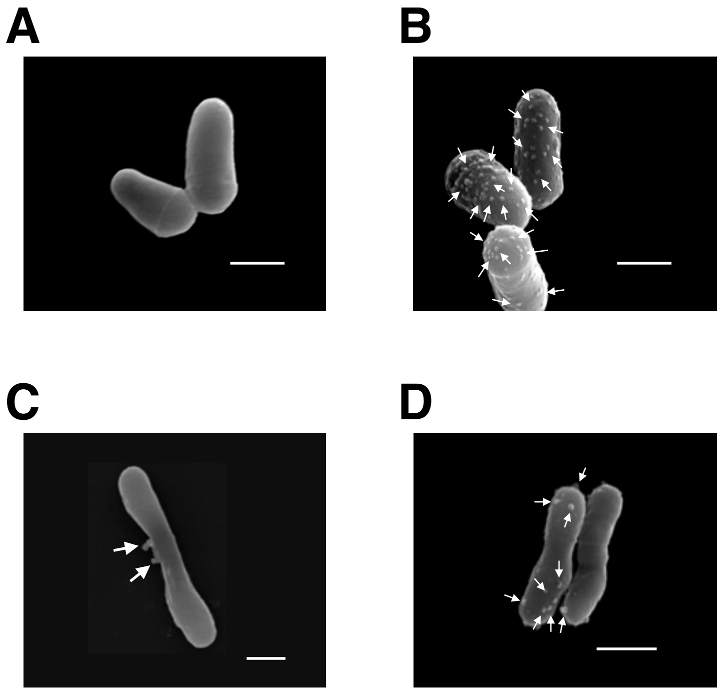

Supplemental Figure 5. Scanning electron microscopy images of *Corynebacterium glutamicum* cells. Related to Fig. 1 and Fig. 2.

C. glutamicum cells under (A) no treatment, (B) biotin-deficient, (C) MMC treatment, and (D) penicillin G treatment conditions were fixed and observed by scanning electron microscopy. White arrows indicate MVs. Scale bars, 1 μm.

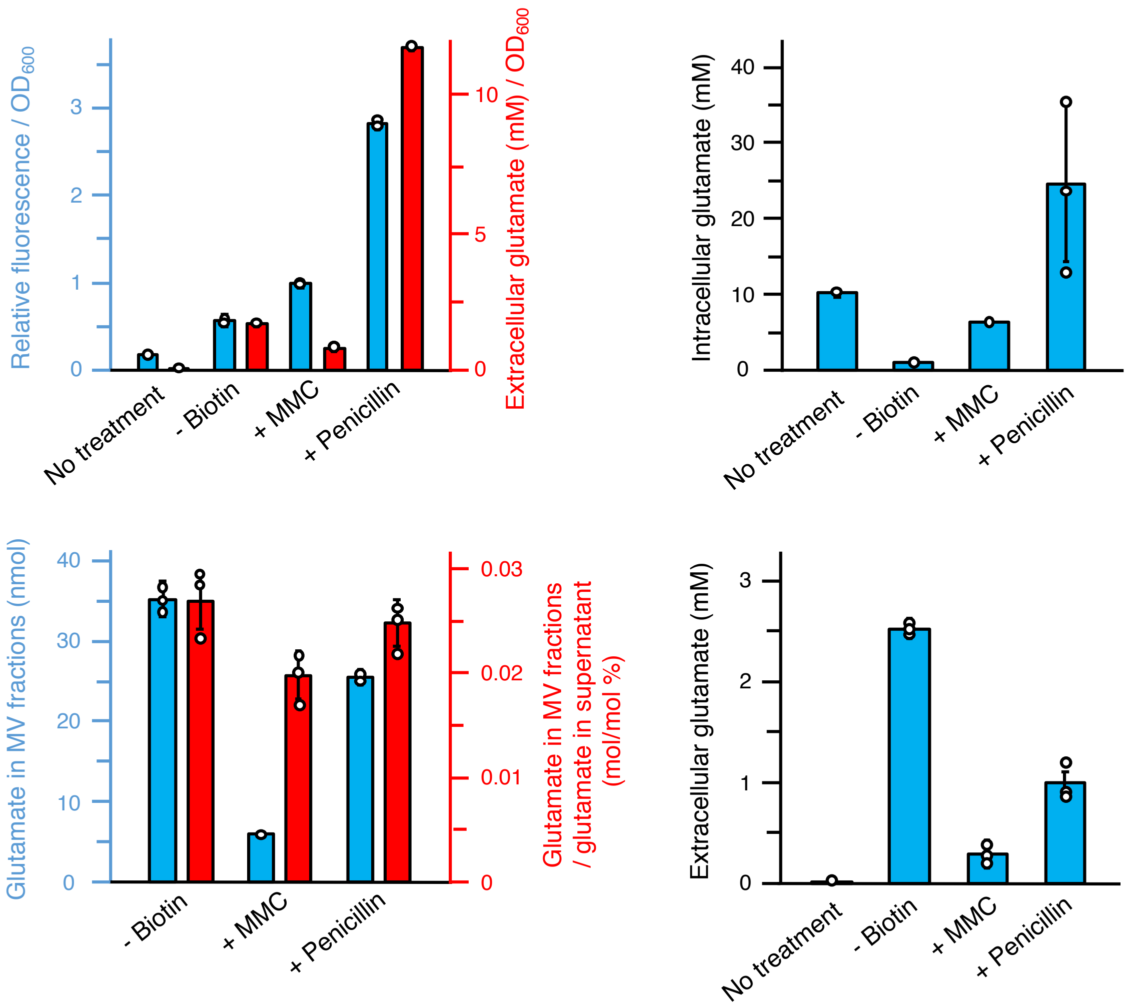

Supplemental Figure 6. Glutamate effluxes of *Corynebacterium glutamicum* under various conditions. Related to Fig. 1 and Fig. 2.

(A) Concentrations of glutamate in the culture media of *C. glutamicum*. (B) MV release (blue) and the concentrations of glutamate in the culture media (red) were normalised to OD_{600}. (C) Intracellular glutamate concentrations of *C. glutamicum* cells that were grown under MV release-inducing conditions. These concentrations were calculated using dried cell weight of the cells. (D) Glutamate was detected and quantified from MVs. Percentages are glutamate in MV fractions to total glutamate in culture supernatant before MV isolation. MVs were purified from 200 mL of the culture medium, and then glutamate was extracted from these MVs (blue). These values were compared with the total amounts of glutamate in the culture supernatant (red). All values indicated by the bars represent the mean value ± S.D. for three experiments.

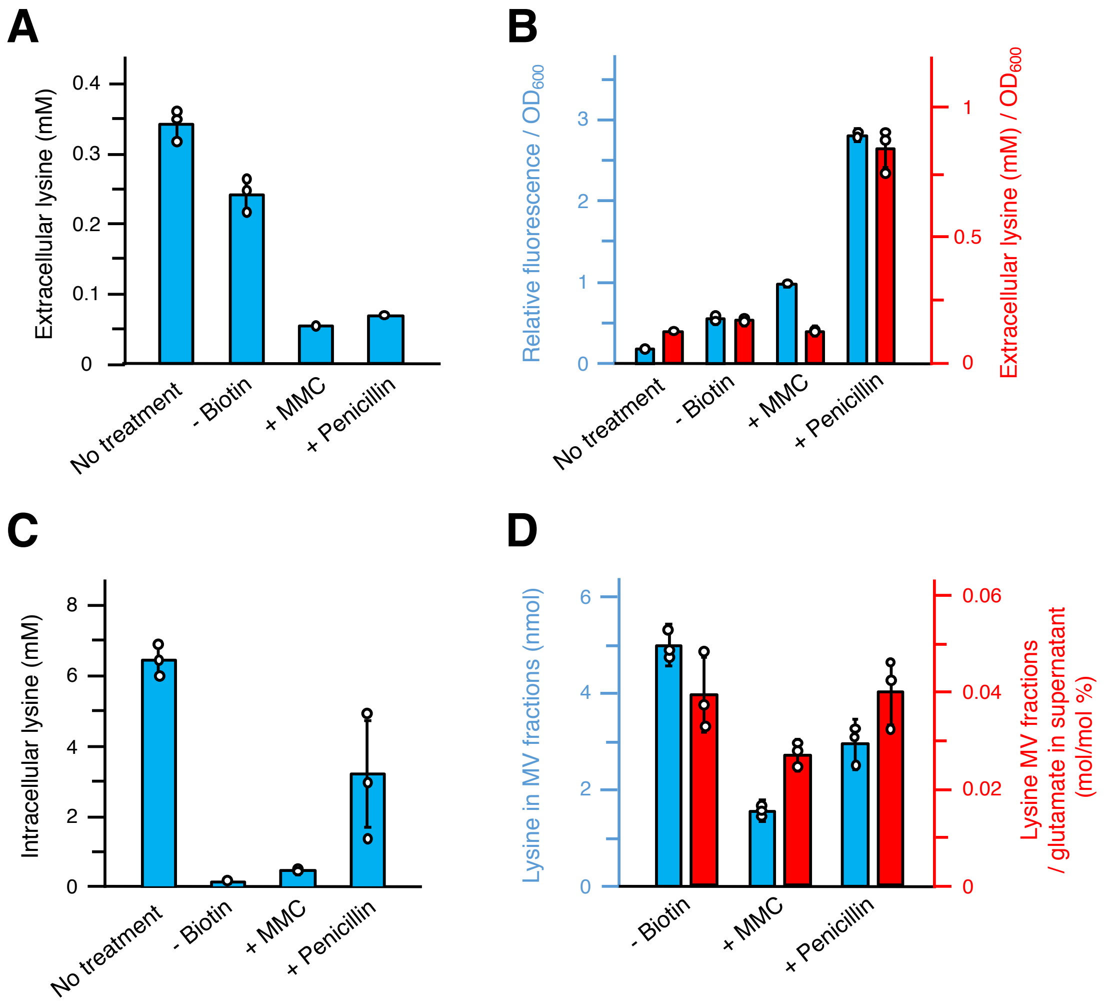

Supplemental Figure 7. Lysine effluxes of *Corynebacterium glutamicum* under various conditions. Related to Fig. 1 and Fig. 2.

(A) Concentrations of lysine in the culture media of *C. glutamicum*. (B) MV release (blue) and the concentrations of lysine in the culture media (red) were normalised to OD_{600}. (C) Concentrations of intracellular lysine of *C. glutamicum* cells that were grown under MV release-inducing conditions. These concentrations were calculated using dried cell weight of the cells. (D) Lysine was detected and quantified from MVs. Percentages are lysine in MV fractions to total lysine in culture supernatant before MV isolation. MVs were purified from 200 mL of the culture medium, and then lysine was extracted from these MVs (blue). These values were compared with the total amounts of lysine in the culture supernatant (red). All values indicated by the bars represent the mean value ± S.D. for three experiments.

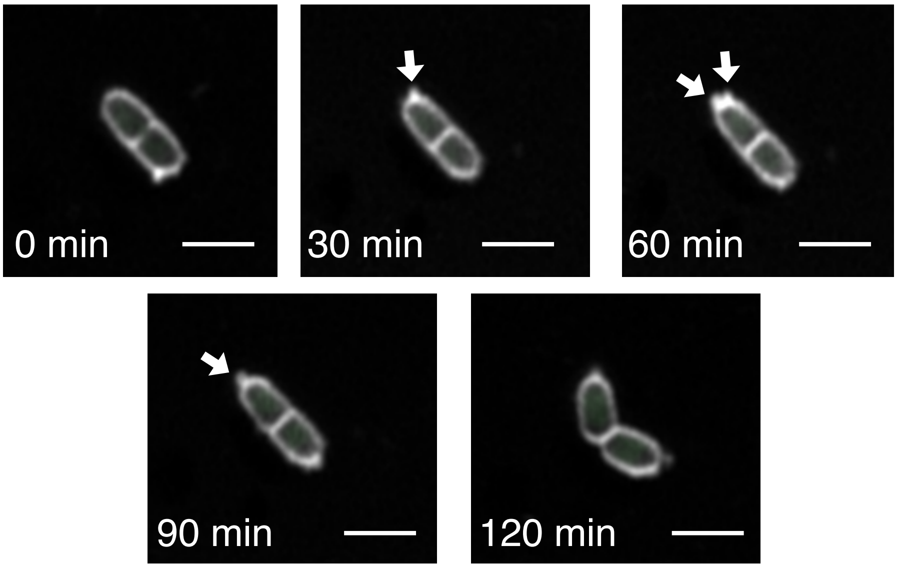

Supplemental Figure 8. Cell division after MV formation in penicillin G condition. Related to Fig. 2.

Cell division of *Corynebacterium glutamicum* cell after MV formation was observed during time-lapse imaging using CLSM in the presence of penicillin G. White arrows indicate MVs. Representative images are shown. White, FM4-64; green, SYTOX green. Scale bar, 2 μm.

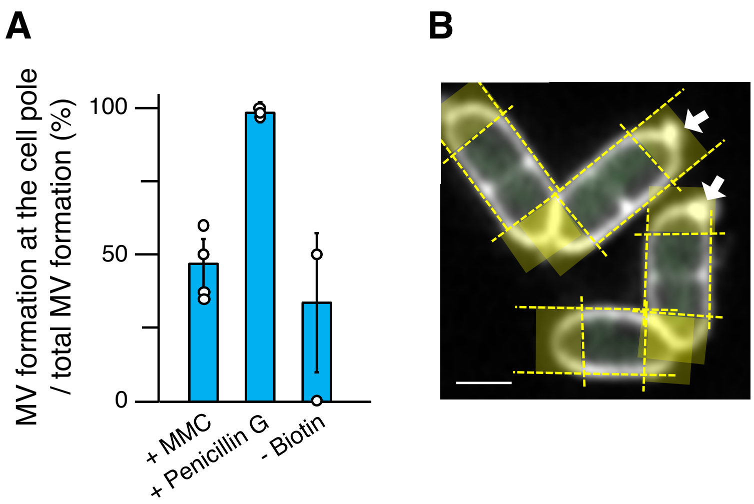

Supplemental Figure 9. MV formation at the cell pole in *Corynebacterium glutamicum*. Related to Fig. 2.

(A) MV formation at the cell pole in *C. glutamicum* was counted using live-cell imaging. *C. glutamicum* was cultured on agarose pad containing MM-1 medium under MMC treatment, penicillin G treatment or biotin-deficient condition. The cells were observed using CLSM for ~5 hours. All values indicated by the bars represent the mean value ± S.D. for 5 fields. (B) A representative image of the cells under penicillin G treatment condition is shown. Curved regions at the end of the rod-shaped cells (yellow regions) were defined as the cell poles under all conditions. White arrows indicate MVs. Scale bar, 1 µm.

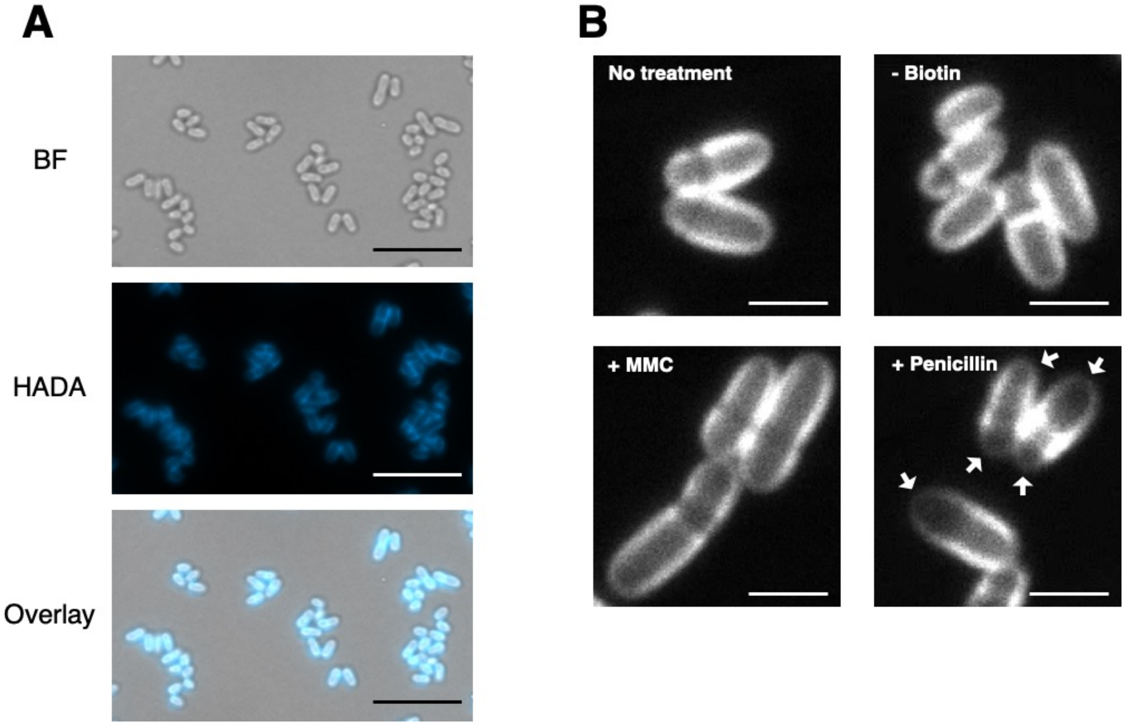

Supplemental Figure 10. Peptidoglycan staining using HADA. Related to Fig. 2.

Peptidoglycans of *Corynebacterium glutamicum* cells were stained by adding HADA to the culture medium. (A) Bright field (BF, top), fluorescence microscopy (middle) and overlay (bottom) of HADA-stained cells under no treatment condition. Scale bars, 10 µm. (B) CLSM images of HADA-stained cells under no treatment or MV formation-inducing conditions. White arrows indicate cell poles where peptidoglycan biosynthesis was inhibited. The images of the cells under normal condition (No treatment) and under penicillin G condition (+Penicillin) were also shown in Fig. 2. Scale bars, 2 µm.

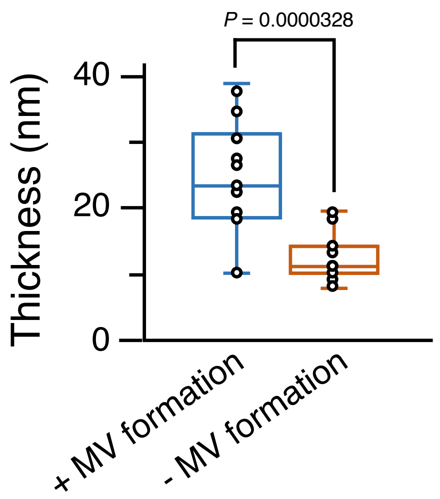

Supplemental Figure 11. MV formation outside of electron-transparent layers of *Corynebacterium glutamicum* cells in biotin deficiency. Related to Fig. 2.

Box plot of thickness of electron-transparent layers (ETLs) in the cell envelope of *C. glutamicum* cells in biotin deficiency. The blue box (left) indicates maximum thickness of the ETLs where MV formations were observed in TEM images of the cells in biotin deficient condition. The orange box (right) indicates thickness of randomly selected ETLs where no MV formation was observed in the same condition. Sample numbers, 14 each. *P* value was calculated using unpaired *t*-test with Welch's correction.

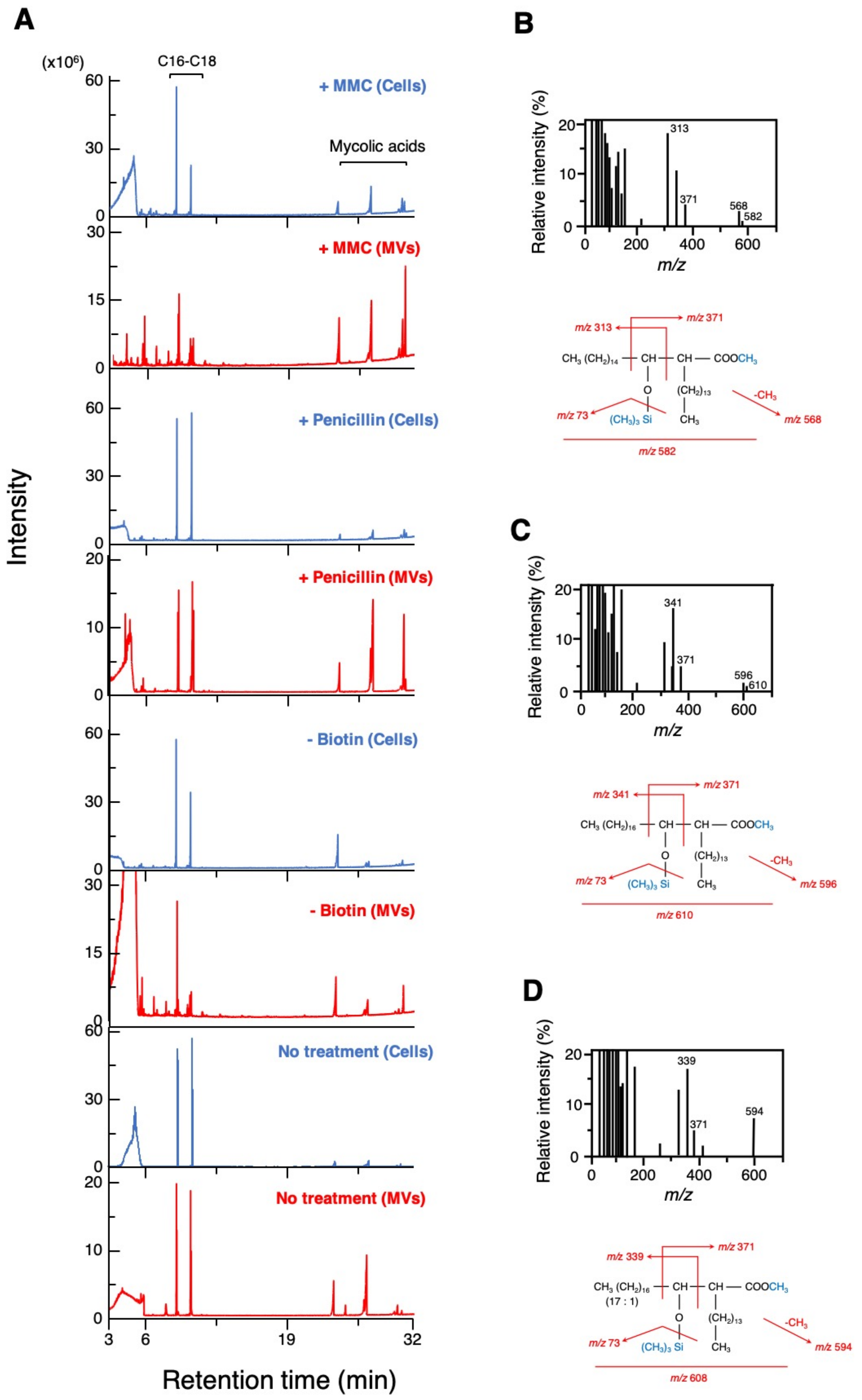
A
(x10⁶)
C16-C18
Mycolic acids
+ MMC (Cells)
+ MMC (MVs)
+ Penicillin (Cells)
+ Penicillin (MVs)
- Biotin (Cells)
- Biotin (MVs)
No treatment (Cells)
No treatment (MVs)
Intensity
Retention time (min)
B
Relative intensity (%)
m/z
313
371
568
582
m/z 313
m/z 371
CH3 (CH2)14 — CH — CH — COOCH3
O
(CH2)13
(CH3)3 Si
CH3
m/z 73
-CH3
m/z 568
m/z 582
C
341
371
596
610
m/z 341
m/z 371
CH3 (CH2)16 — CH — CH — COOCH3
m/z 73
-CH3
m/z 596
m/z 610
D
339
371
594
m/z 339
m/z 371
CH3 (CH2)16 — CH — CH — COOCH3
(17 : 1)
m/z 73
-CH3
m/z 594
m/z 608

Supplemental Figure 12. Structures of corynomycolic acids that were detected in *Corynebacterium glutamicum* cells and MVs. Related to Fig. 3.

(A) Detection of fatty acids by gas chromatography/mass spectrometry (GC/MS). The lipids were extracted from lyophilised cells and MVs. C16-C18 fatty acids and C32-C34 corynomycolic acids were detected as methylester and methylester-trimethylsilyl derivatives, respectively. (B-D) The structures of corynomycolic acids were determined based on the fragmentation patterns on GC/MS analyses and a previous study (Hashimoto et al, 2006).

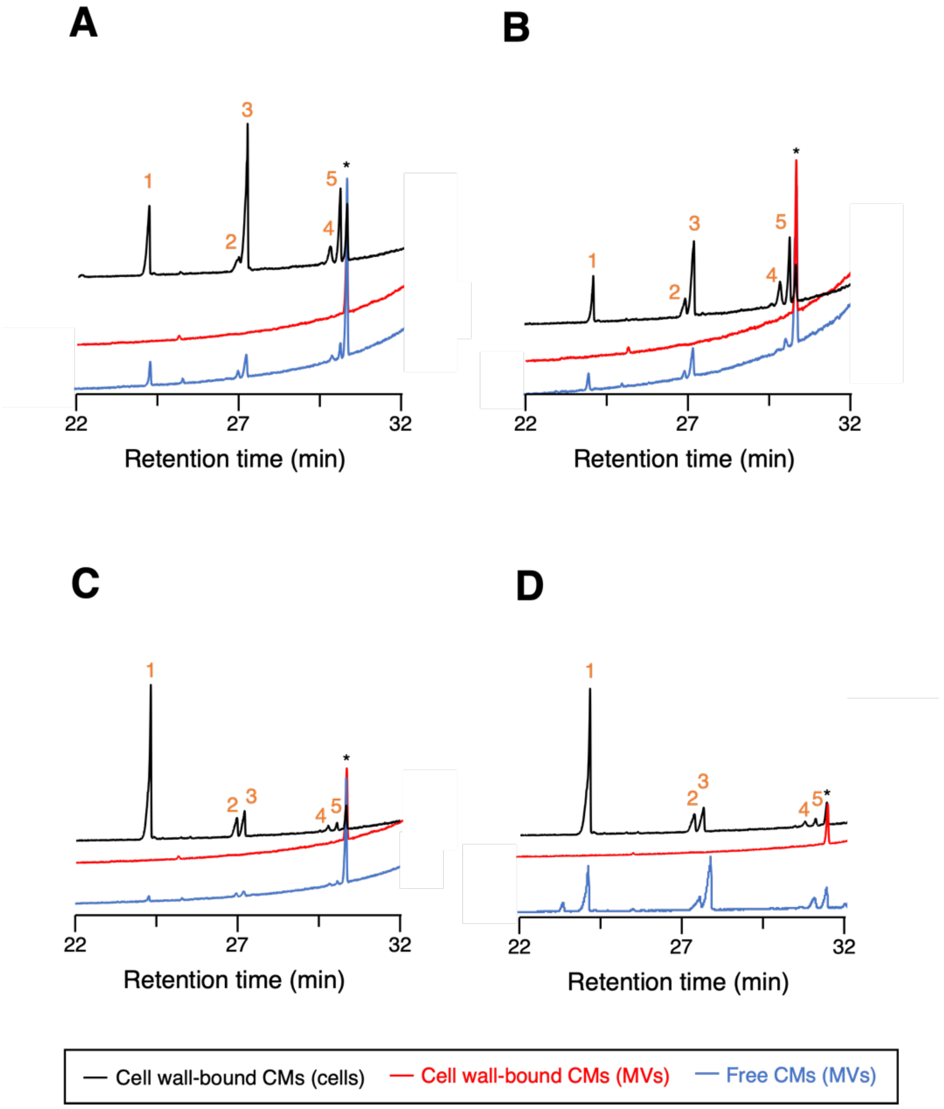

Supplemental Figure 13. GC/MS analyses of corynomycolic acids that are free or covalently bound to cell wall. Related to Fig. 3.

Corynomycolic acids (CMs) were extracted from cell walls and analysed by GC/MS. Cell wall-bound CMs were extracted and derivatised by hydrolysis of corynomycolic acids (CM)-arabinogalactan-peptidoglycan complex from the cells and MVs. Free CMs were sequentially extracted from MVs using MeOH: $CHCl_3$ = 2:1, 1:1 and 1:2 solutions. Cells were grown under (A) MMC treatment, (B) penicillin treatment and (C) biotin-deficient and (D) no treatment conditions, and the corresponding MVs were purified. Each number indicates the following CMs: 1, C32 CM (*m/z* 582); 2, C34:1 CM (*m/z* 608); 3, C34 CM (*m/z* 610); 4 and 5, undetermined CMs that exhibit *m/z* values of 638 and 636, respectively. The structures of these CMs were determined in Supplemental Fig. 12. Asterisk, solvent-derived peaks.

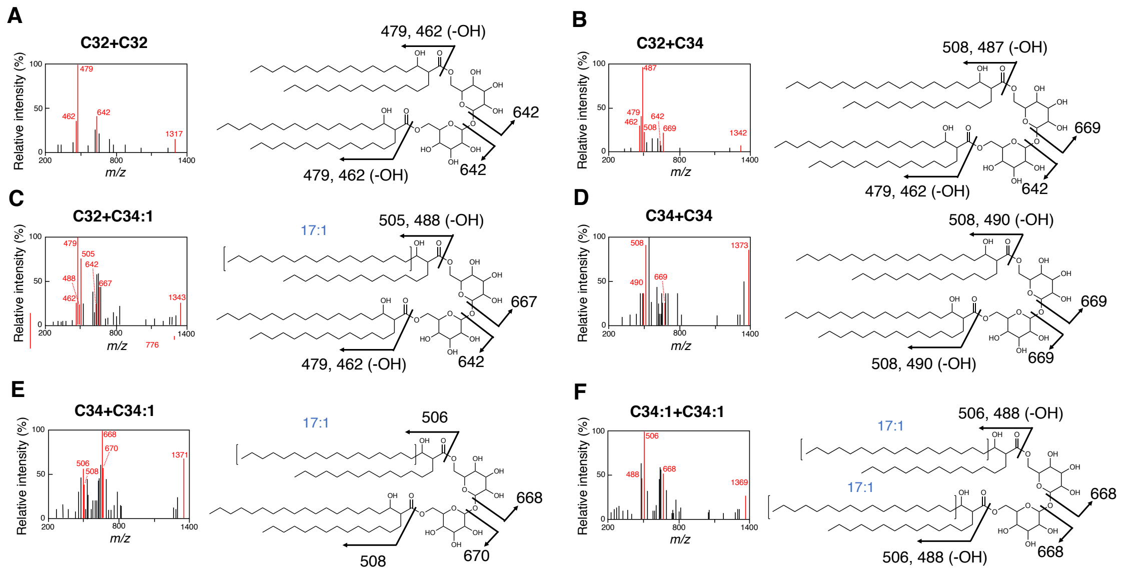

Supplemental Figure 14. Detection of trehalose dicorynomycolic acids from MVs. Related to Fig. 3.

Trehalose dicorynomycolic acids (a-f, TDCMs) were detected from MVs using LC/MS/MS. Representative results are shown. These molecules were searched from the lipid extracts of MVs based on the results of GC/MS analyses.

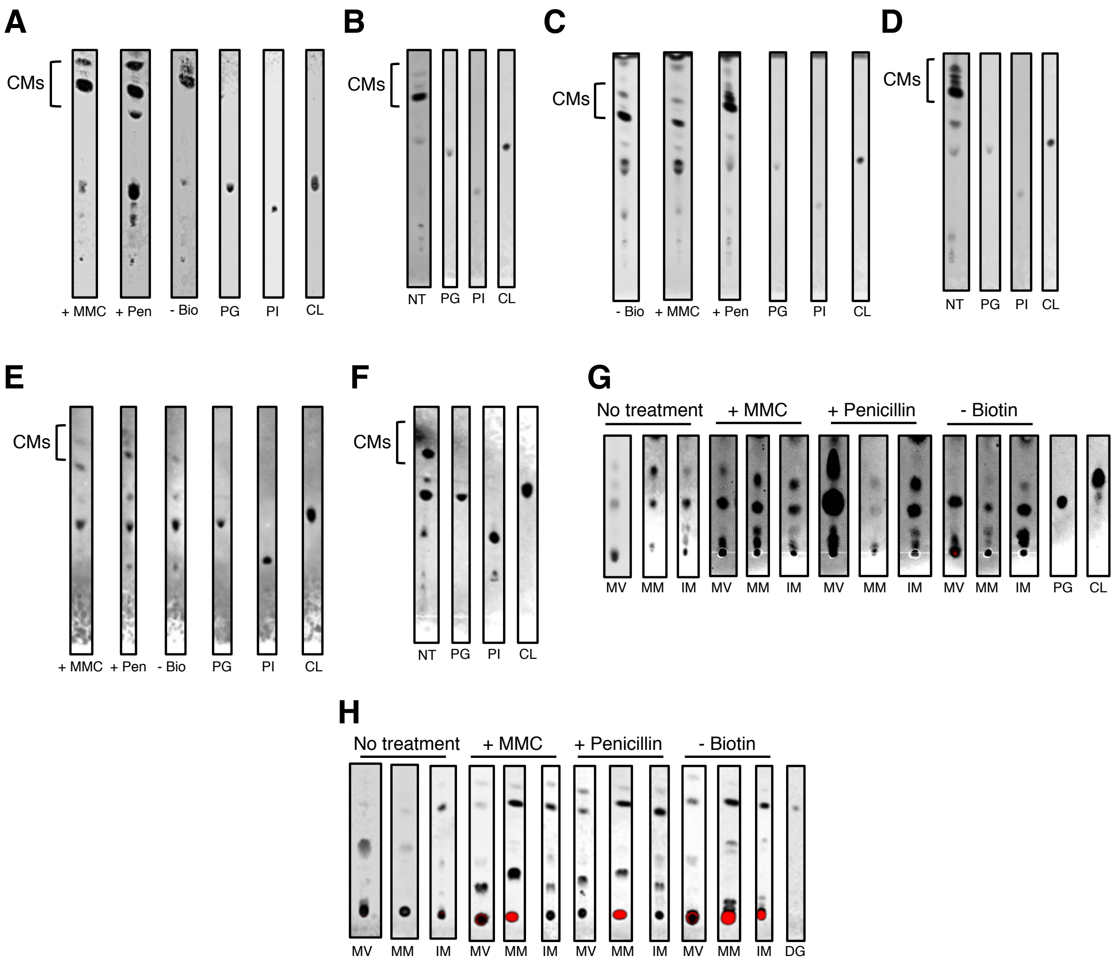

Supplemental Figure 15. Thin-layer chromatography profiles of *Corynebacterium glutamicum* cells and MVs. Related to Fig. 3.

Major lipids in cells and MVs were detected by thin-layer chromatography (TLC). Phosphatidylglycerols (PGs), phosphatidylinositols (PIs), cardiolipins (CLs) and corynomycolic acids (CMs) in (A, B) MVs fractions, (C, D) cellular mycomembrane fractions and (E, F) cellular inner membrane fractions were separated using chloroform:methanol:H_2O = 65:25:4 (v/v) as a solvent. NT, no treatment condition; +MMC, MMC treatment condition; +Pen, penicillin G treatment condition; -Bio, biotin-deficient condition. (G) PG and CL were separated using chloroform: hexane: methanol: acetate = 50:30:10:5 (v/v) as a solvent. (H) Diacylglycerols (DG) were separated using toluene: chloroform: acetone = 7:2:1 (v/v) as a solvent. L-α-phosphatidyl-DL-glycerol (distearoyl), phophatidylinositol, cardiolipin from bovine heart and 1,2-dipalmitoyl-*rac*-glycerol were used as standards. All lipids were labelled with primuline and detected at 365 nm.

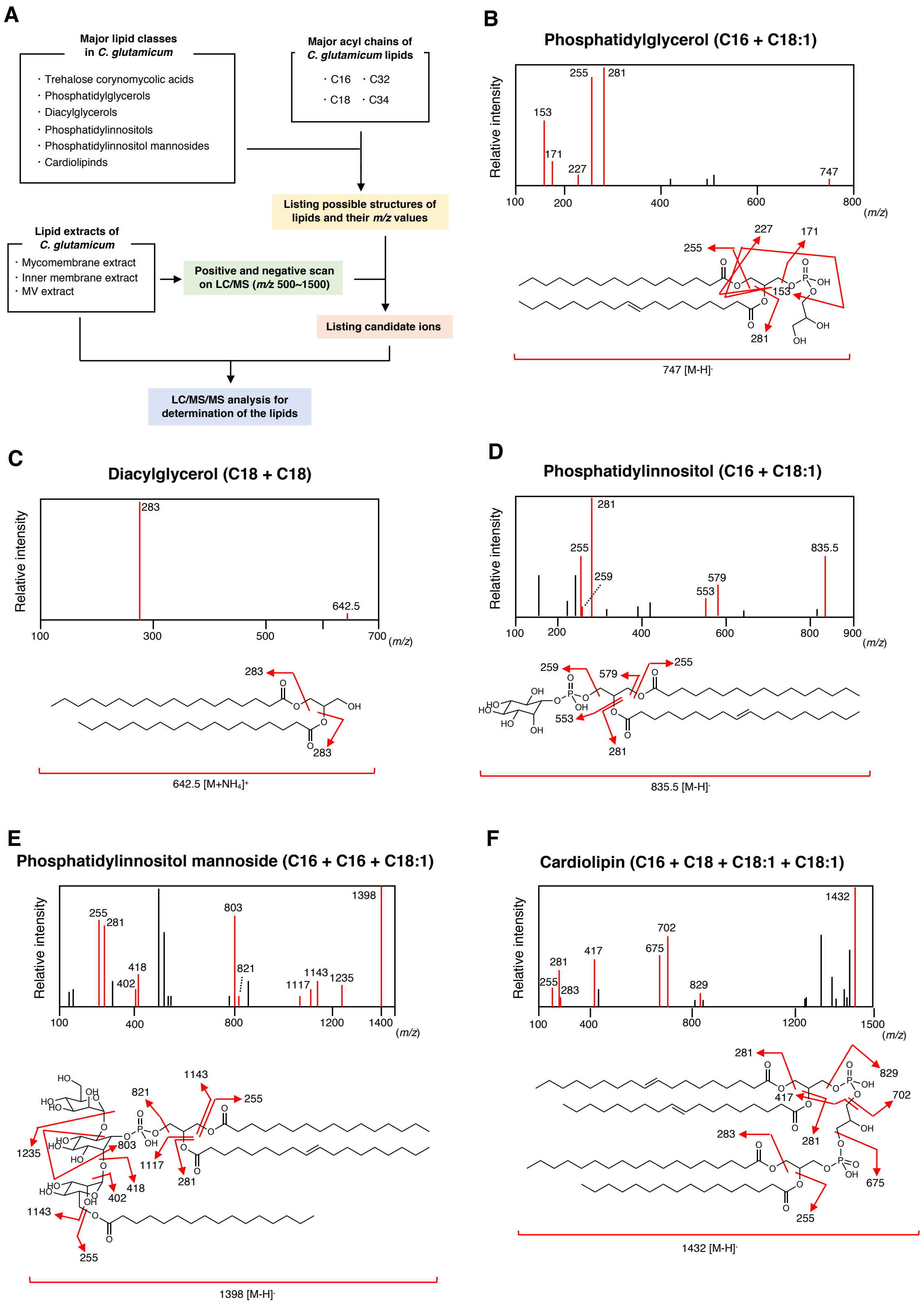
A
Major lipid classes in C. glutamicum
· Trehalose corynomycolic acids
· Phosphatidylglycerols
· Diacylglycerols
· Phosphatidylinnositols
· Phosphatidylinnositol mannosides
· Cardiolipinds
Major acyl chains of C. glutamicum lipids
· C16 · C32
· C18 · C34
Listing possible structures of lipids and their m/z values
Lipid extracts of C. glutamicum
· Mycomembrane extract
· Inner membrane extract
· MV extract
Positive and negative scan on LC/MS (m/z 500~1500)
Listing candidate ions
LC/MS/MS analysis for determination of the lipids
B
Phosphatidylglycerol (C16 + C18:1)
Relative intensity
747 [M-H]-
C
Diacylglycerol (C18 + C18)
Relative intensity
642.5 [M+NH4]+
D
Phosphatidylinnositol (C16 + C18:1)
Relative intensity
835.5 [M-H]-
E
Phosphatidylinnositol mannoside (C16 + C16 + C18:1)
Relative intensity
1398 [M-H]-
F
Cardiolipin (C16 + C18 + C18:1 + C18:1)
Relative intensity
1432 [M-H]-
(m/z)

Supplemental Figure 16. Identification of major lipids in *Corynebacterium glutamicum*. Related to Fig. 3.

Lipids of *C. glutamicum* were detected and determined. Schematic diagram of the determination of the lipids is shown in *A*. Major classes of the lipids in *C. glutamicum* are determined based on previous studies (Brennan et al, 1995; Klatt et al, 2018) and our thin-layer chromatography analyses (Supplemental Fig. 15). Based on our results of GC/MS analysis indicating that acyl chain lengths of major lipids in *C. glutamicum* under the tested condition were C16, C18, C32 and C34 (Supplemental Fig. 12), we listed the possible structures of the major lipids which include these fatty acids. We then analysed the lipid extracts of *C. glutamicum* (described under Methods) on LC/MS in the scanning mode. In this analysis, we searched ions whose *m/z* values correspond to those of the listed lipids and then listed these ions as candidate ions derived from *C. glutamicum* lipids. Structures of the candidate ions were determined by LC/MS/MS analysis. (B-F) Representative results of LC/MS/MS analysis of the lipids are shown. Structures of the lipids containing C16 and/or C18 fatty acids were determined based on *m/z* values of their fragment ions and the fragmentation patterns. The fragmentation patterns and the determined structures are consistent with those of previous studies (Cox et al, 2009; Hsu et al, 2001, 2007; Kind et al, 2013; Minkler et al, 2010; Mishra et al, 2009). Characteristics of each class of lipids in the LC/MS/MS analysis are as follows. Phosphatidylglycerol (B) is characterised by $[M\text{-}H]^-$ precursor ion and the fragment ions derived from fatty acid chains and phosphate. Diacylglycerol (C) is characterised by $[M+NH_4]^+$ precursor ion and the fragment ions derived from fatty acid chains. Phosphatidylinnositol (D) is characterised by $[M\text{-}H]^-$ precursor ion and the fragment ions derived from fatty acid chains and inositol phosphate. Phosphatidylinnositol mannoside (E) is characterised by $[M\text{-}H]^-$ precursor ion, the fragment ions derived from fatty acid chains and innositol phosphate, and neutral loss of a mannoside moiety. Cardiolipin (F) is characterised by $[M\text{-}H]^-$ precursor ion and the fragment ions derived from fatty acid chains and two phosphatidylglycerol moiety. Details of the analyses are described under Methods. Determined structures of trehalose dicorynomycolic acids containing C32 and/or C34 acyl chains are shown in Supplemental Fig. 14.

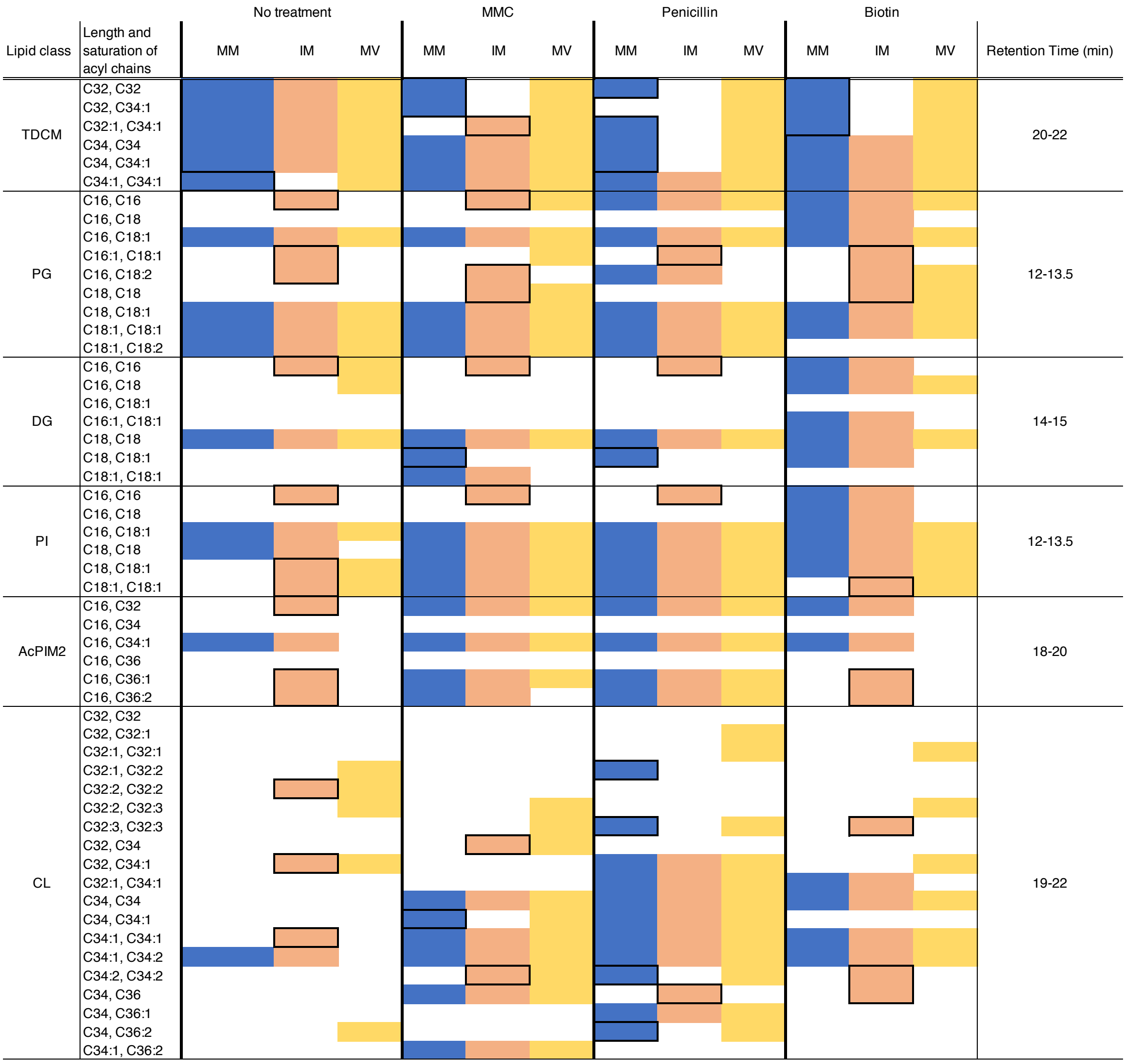

Supplemental Figure 17. Catalogue of the membrane lipids of *Corynebacterium glutamicum*. Related to Fig. 3.

Major lipids in *C. glutamicum* were analysed and listed. Detail of the analysis is described under Methods and Supplemental Fig. 16. The detected lipids are indicated as blue (mycomembrane, MM), orange (inner membrane, IM) or yellow (MV fraction). The lipids that were detected exclusively in mycomembrane or inner membrane are indicated by black bold squares. TDCM, trehalose dicorynomycolic acid; PG, phosphatidylglycerol; DG, diacylglycerol; PI, phosphatidylinnositol; AcPIM2, acylated phosphatidylinnositol mannoside; CL, cardiolipin.

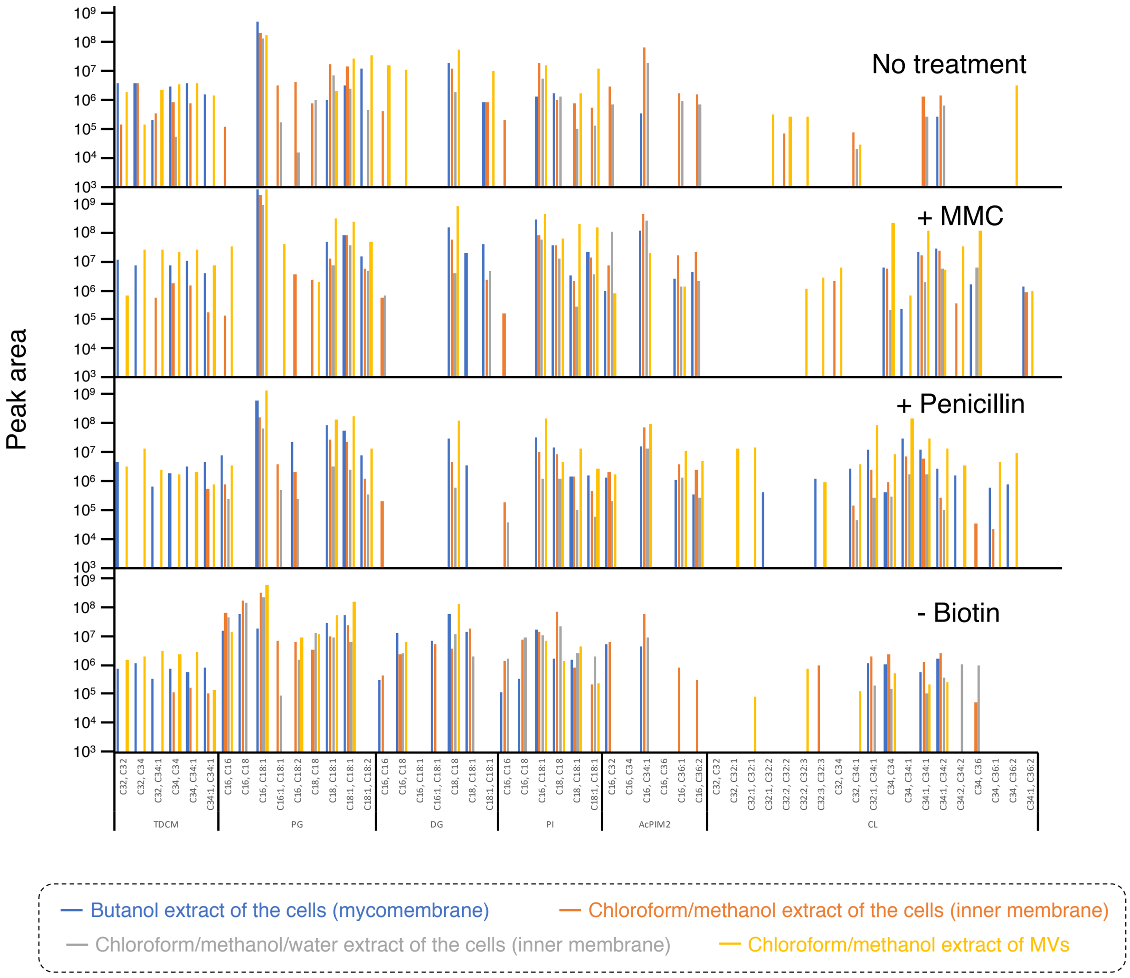

Supplemental Figure 18. Intensities of the membrane lipids of *Corynebacterium glutamicum* in LC/MS analysis. Related to Fig. 3.

Lipid compositions of MVs were analysed by LC/MS. Detail of the analysis is described under Methods. Major lipids in *Corynebacterium glutamicum* were detected and identified based on fragmentation patterns of the lipids on LC/MS/MS analysis (Supplemental Fig. 16) and the previous studies (Cox et al, 2009; Hsu et al, 2001, 2007; Kind et al, 2013; Minkler et al, 2010; Mishra et al, 2009). Average values of three independent experiments are shown. PG, phosphatidylglycerol; DG, diacylglycerol; PI, phosphatidylinositol; AcPIM2, acyl phosphatidyl-*myo*-inositol dimannoside; CL, cardiolipin.

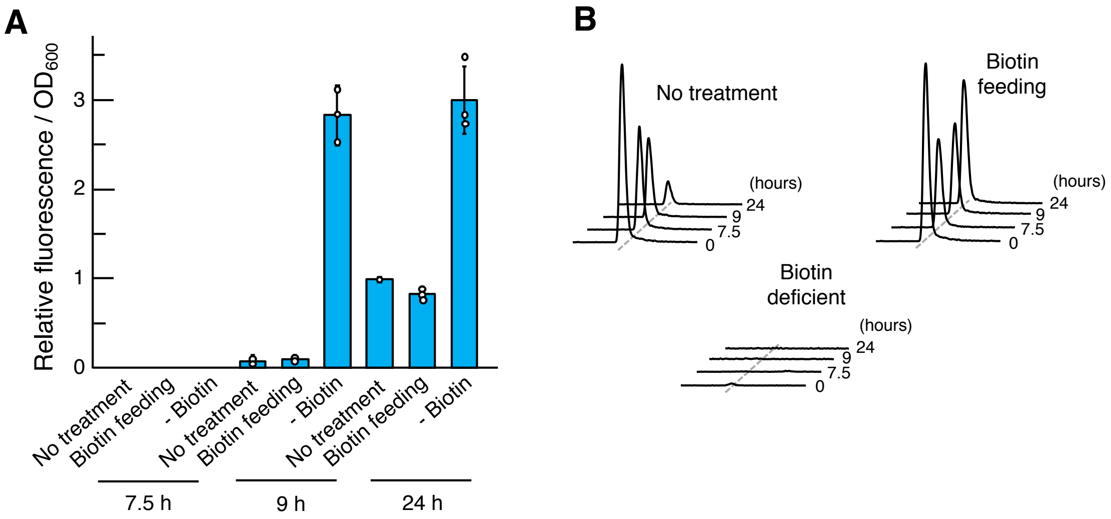

Supplemental Figure 19. *Corynebacterium glutamicum* releases MVs even under biotin-sufficient condition. Related to Fig. 3.

(A) MVs were isolated and quantified from the culture media at each time point during cultivation. No treatment and biotin-deficient (-Biotin) conditions contain 200 and 1 μg L^{-1} biotin, respectively. In biotin-feeding condition, biotin (200 μg L^{-1}) was added to the culture medium of no treatment condition after 9 h of cultivation. All values indicated by the bars represent the mean value ± S.D. for three experiments. (B) Biotin was detected from each culture medium by LC/MS as a product ion which exhibits *m/z* value of 227 in the positive ion mode. Representative chromatograms were shown.

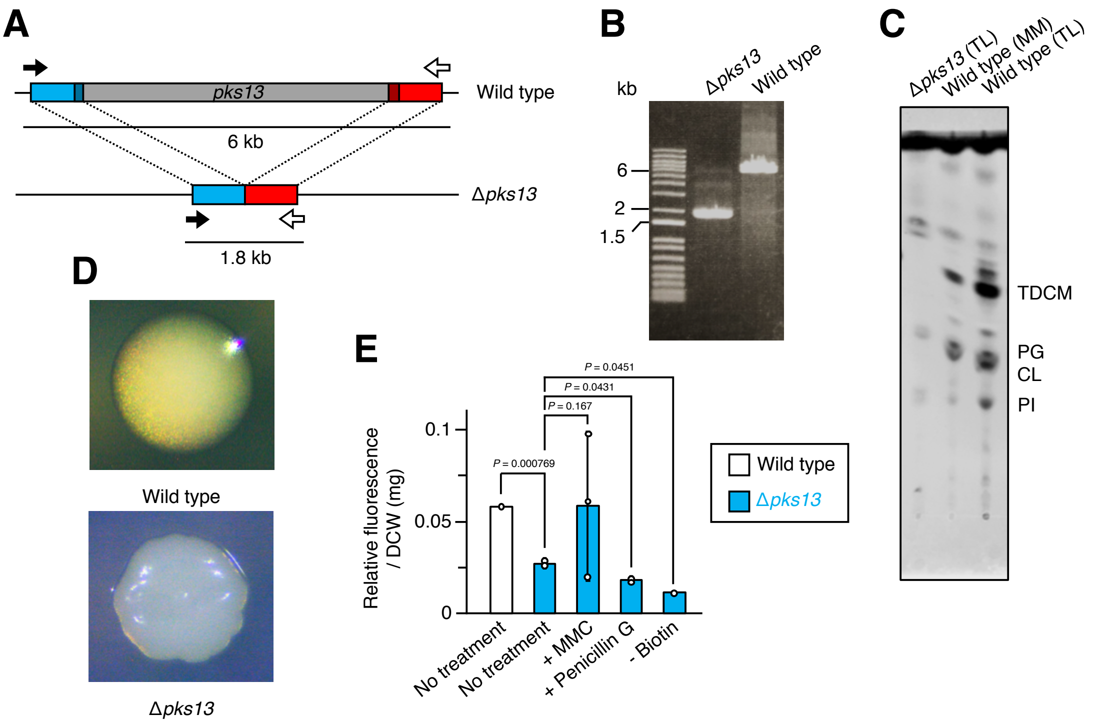

Supplemental Figure 20. MV release by Δ*pks13* mutant of *Corynebacterium glutamicum*. Related to Fig. 3.

(A) Schematic diagram of deletion of *pks13* gene coding a polyketide synthase (PKS13) which catalyzes the last condensation step in the biosynthesis of CMs (Portevin et al, 2004). Arrows indicate the primer set (PKS13_Check_Fw and PKS13_Check_Rv) used for PCR in *B.* (C) TLC analysis of the lipids from wild type and Δ*pks13* mutant of *C. glutamicum*. The lipids were separated using chloroform: methanol: H_2O = 65:25:4 (v/v) as a solvent. TL, total lipids; MM, mycomembrane. (D) Colony morphology of wild type and Δ*pks13* mutant of *C. glutamicum*. (E) MV release of wild type and Δ*pks13* mutant of *C. glutamicum*. Growth conditions were described under Methods. MVs were quantified using FM4-64 dye, and the relative fluorescence were normalised to dried cell weight (DCW). All values indicated by the bars represent the mean value ± S.D. for three experiments. *P*-values were calculated using *t*-test with Welch's correction.

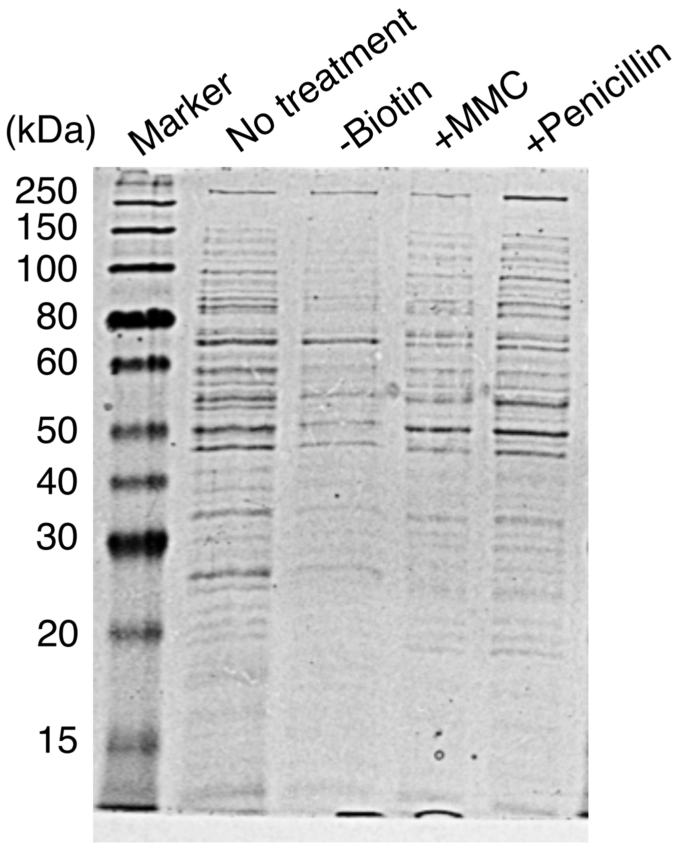

Supplemental Figure 21. SDS-PAGE of *Corynebacterium glutamicum*-derived proteins. Related to Fig. 4.

SDS-PAGE profiles of whole-cell proteins of *C. glutamicum* are shown. After cultivation of *C. glutamicum* in biotin deficiency and under MMC or penicillin G conditions, whole-cell proteins were obtained from the cells by sonication, and subjected to SDS-PAGE. Five micrograms of proteins were applied to each lane.

```
  1 MNKLATRALVALTGSAIAMTGLTVVSANAA
 31 EKTGKCRVVTTTGTADWSVRESFNNYLEGP
 61 IANGAAYKYHGGIEVRDGVETTGTKSAREF
 91 TWPVLGSEEGAVKLGGGVHWTGHNHYSGDD
121 ESQAPDNFILDLDFSNPTVKFDGNEGTLLV
151 DFKSREFVDTKTVADFLTGTQAELATITFD
181 EPIDLTQENVTVTGQTKLTATGVDVMGTFY
211 PEGEALAPITLNLTNEVVCDEPETPVEPEV
241 PVEPETPVDPETSVDPETPVDPETSVDPEK
271 PGDDNKDDGSNSSSNGDILGILGILAALGG
301 VGALVYNFLVASGFLAAFK
```

Signal peptide   Transmembrane helix

Supplemental Figure 22. Amino acid sequence of NCgl0381. Related to Fig. 4.

Amino acid sequence of NCgl0381 was analysed using SignalP 5.0 (Armenteros et al, 2019) and SOSUI (Mitaku et al, 2002). Blue and green regions indicate the deduced signal peptide and transmembrane helix, respectively, in NCgl0381.

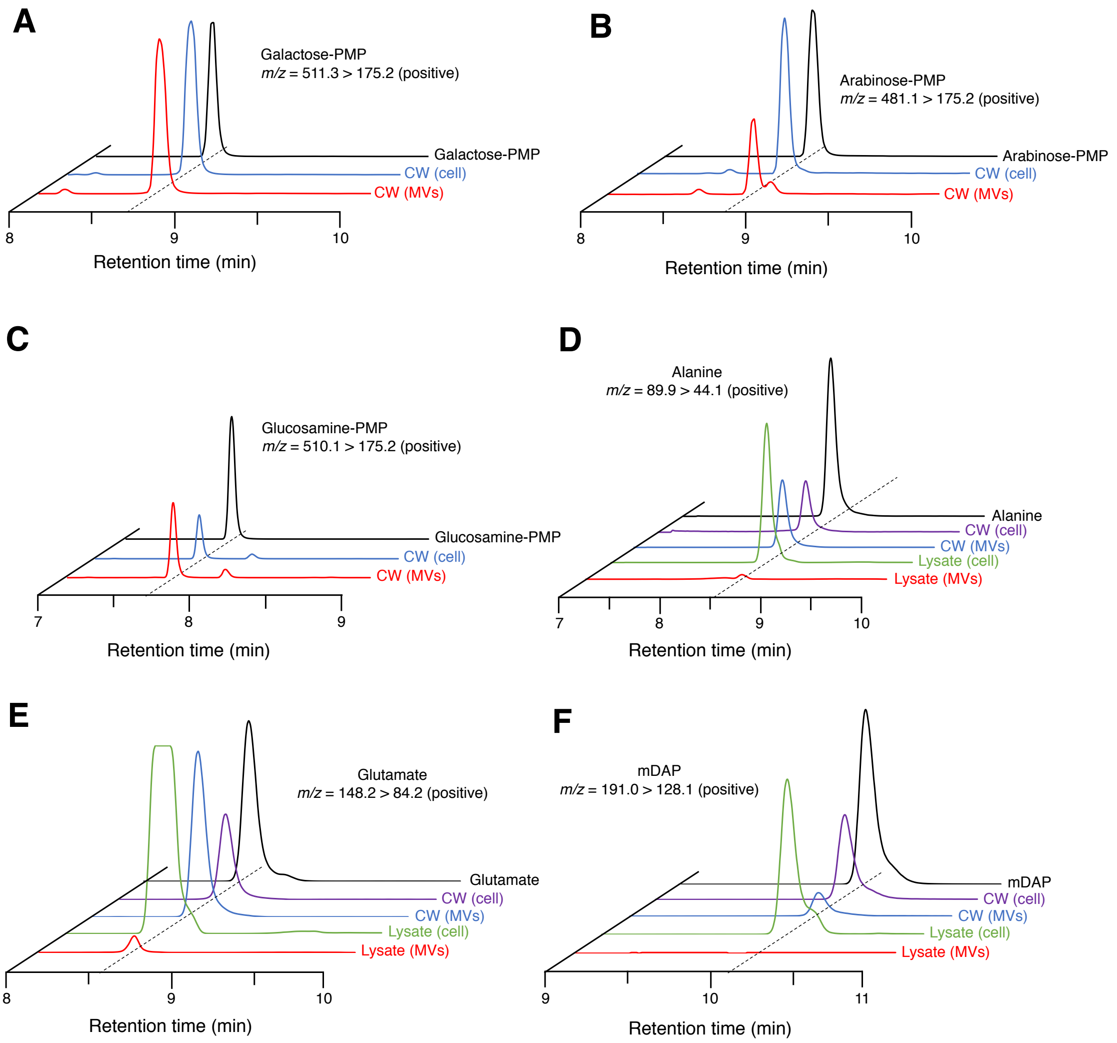

Supplemental Figure 23. Detection of cell wall components from M-MVs. Related to Fig. 4.

Cell wall fragments (CW) were purified from M-MVs and then hydrolysed. (A, B) Arabinogalactan and (C-F) peptidoglycan components were detected by multiple reaction monitoring (MRM) mode using LC/MS. Sugars were derivatised with 3-methyl-1-phenyl-5-pyrazolone (PMP) and then subjected to the analyses.

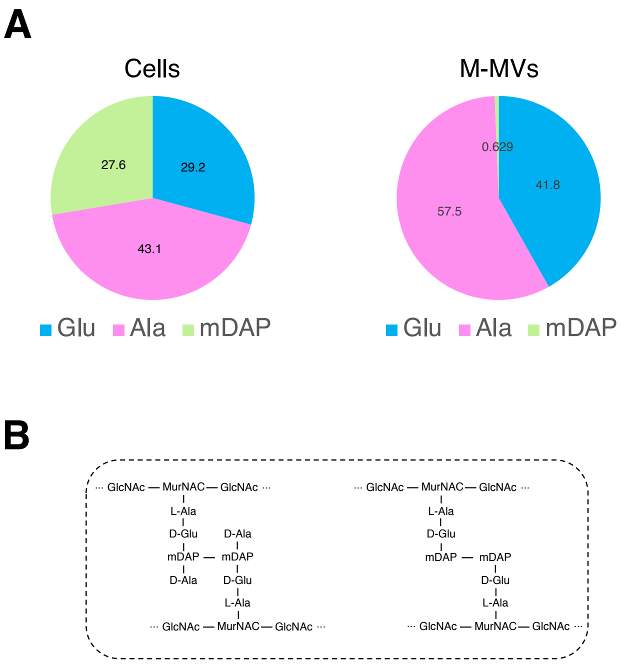

Supplemental Figure 24. Compositions of amino acids of peptidoglycan in *Corynebacterium glutamicum* cells and M-MVs. Related to Fig. 4.

(A) Molar ratio of amino acids of purified peptidoglycan were calculated based on the results of LC/MS analyses. The peptidoglycan was purified from MMC-treated cells and M-MVs. (B) Two types of peptide bridges are present in the peptidoglycan of *C. glutamicum* (Bukovski, 2013).

Supplemental Table 1. Bacterial strains used in this study. Related to Fig. 1 to Fig. 5.

Bacterial strains used in this study and their characteristics are shown.

Strains	Characteristics
Corynebacterium glutamicum ATCC13032 (NBRC12168)	Wild-type strain
Corynebacterium glutamicum Δ*NCgl1682*	Δ*NCgl1682* mutant of wild-type strain
Corynebacterium glutamicum Δ*pks13*	Δ*pks13* mutant of wild-type strain
Mycobacterium smegmatis MC^2155	Wild-type strain
Rhodococcus erythropolis PR4	Wild-type strain
Rhodococcus equi IFO3730	Wild-type strain
Escherichia coli DH5α	F^-, Φ80d*lacZ*ΔM15, Δ(*lacZYA-argF*)U169, *deoR*, *recA*1, *endA*1, *hsdR*17(r_K^-, m_K^+), *phoA*, *supE*44, $λ^-$, *thi*1, *gyrA*96, *relA*1

Supplemental Table 2. Primers and plasmids used in this study. Related to Fig. 1 to Fig. 3.

(A) Names and nucleotide sequences of primers used in this study are shown. Cleavage sites for restriction enzymes are underlined. (B) Names and characteristics of plasmids used in this study are shown.

A

Primers	Sequences
NCgl1682_FR1_Fw (EcoRI)	TATGACCATGATTACGAATTTCCCTCTAAACAAAACCACCAATTC
NCgl1682_FR1_Rv	AAGGTAAAGGACAAACGAACTGTAGTGGTAGAGGGTTAAAAAAC
NCgl1682_FR2_Fw	GTTCGTTTGTCCTTTACCTTTAC
NCgl1682_FR2_Rv (HindIII)	ACGACGGCCAGTGCCAAGCTTCAATGGAGAATTTTGGACC
NCgl1682_Ex_Fw (PstI)	CCAAGCTTGCATGCCGAACTGGGTGTAGTGATTTCTGTT
NCgl1682_Ex_Rv (BamHI)	GAGCTCGGTACCCGGGGATCAAAAAACAGCCATCAACATAACGTT
PKS13_FR1_Fw (EcoRI)	TATGACCATGATTACGAATTTCACAGGTGCATACACTGCG
PKS13_FR1_RV	GAATGCTGTCCATGATGACCGA
PKS13_FR2_Fw	GGTCATCATGGACAGCATTCGGAGGTGTCCAGCAGGTTTTC
PKS13_FR2_Rv (HindIII)	ACGACGGCCAGTGCCAAGCTCGACGATGCAGCTGCTGAAG
PKS13_Check_Fw	TCTGCGGAAGTGATCTGCTCGCC
PKS13_Check_Rv	GGCTGCTTCCGCACACATCCG

B

Plasmids	Characteristics
pK18mobSacB	A plasmid for gene deletion in *C. glutamicum*, KanR
pVK7	Shuttle vector for *C. glutamicum* and *E. coli*, KanR

Transparent Methods

Materials

LB medium, FM4-64, PicoGreen, and SYTOX green were purchased from Life Technologies (Carlsbad, CA, USA). Mitomycin C (MMC) and penicillin G were purchased from Nacalai Tesque Co., Inc. (Kyoto, Japan). (+)-Biotin and phophatidylinositol were purchased from FUJIFILM Wako Pure Chemical Corporation (Osaka, Japan). L-α-phosphatidyl-DL-glycerol (distearoyl) was purchased from Tokyo Kasei Kogyo, Co, Ltd. (Tokyo, Japan). Cardiolipin from bovine heart was purchased from Olbracht Serdary Research Laboratories (Toronto, Canada). 1,2-Dipalmitoyl-*rac*-glycerol was purchased from Merck (Darmstadt, Germany). All other chemicals used were from commercial sources and of analytical grade. *Corynebacterium glutamicum* NBRC 12168 (ATCC 13032) was supplied by National Institute of Technology and Evaluation (Chiba, Japan).

Bacterial strains and plasmids

Strains used in this study are listed in Table S1. Primers and plasmids used in this study are listed in Table S2.

The NCgl1682 deletion strain of *C. glutamicum* was constructed as follows. The *NCgl1682* upstream and downstream regions were amplified by PCR using NCgl1682_FR1_Fw/NCgl1682_FR1_Rv and NCgl1682_FR2_Fw/NCgl1682_FR2_Rv primer sets, respectively. The PCR products were integrated with pK18mobSacB by In-Fusion (Clontech Laboratories Inc., CA, USA). The resulting plasmid was transformed into *C. glutamicum* as follows (van der Rest et al, 1999). *C. glutamicum* was cultured in LB medium supplemented with 2% glucose (w/v) at 30 ºC. After culturing overnight, *C. glutamicum* was inoculated (OD_{600} = 0.2) in 100 mL of Epo medium containing 1 g tryptone, 0.5 g yeast extract, 1 g NaCl, 400 mg isoniazide, 2.5 g glycine and 0.1 mL of Tween 40 per 1 L. *C. glutamicum* was then cultured for 24 h at 18 ºC. The shaking speed was 120 rpm. After cultivation, the cells were collected by centrifugation (4,000 × *g*, 10 min). The cells were washed five times with 10% glycerol (v/v). Finally, the cells were resuspended in 500 μL of 10% glycerol. In total, 100 μL of the solution was used for electroporation. Electroporation was performed under the following conditions: 25 μF, 600 kΩ, and 2.5 kV. In total, 1 mL of brain-heart infusion medium supplemented with 91 g L^{-1} sorbitol was immediately added to the solution. The resulting solution was incubated at 46 ºC for 6 min. After heat shock, the solution was incubated for 30 ºC for 1 h. Transformants and deletion mutant were selected with 25 μg/mL kanamycin and 10% (w/v) sucrose, respectively. For complementation of NCgl1682, *NCgl1682* and upstream region (150 bp) were amplified by PCR using NCgl1682_Ex_Fw/NCgl1682_Ex_Rv primer set, and integrated with pVK7 vector by In-Fusion. The resulting plasmid was transformed into *C. glutamicum* as described above. Transformants were selected with 25 μg mL^{-1} kanamycin.

Culture conditions

All bacteria were preincubated in LB medium. After preincubation at 30ºC for 24 h and washing cells, *C. glutamicum* was inoculated in 100 mL of minimum medium (MM-1) consisting 80 g glucose, 30 g $(NH_4)_2SO_4$, 5.7 g Na_2SO_4•$7H_2O$, 6 g KH_2PO_4, 2 g NaCl, 3.9 mg $FeCl_3$, 1.77 mg $ZnCl_2$, 0.44 mg $CuSO_4$•$5H_2O$, 5.56 mg $(NH_4)_6Mo_7O_{24}$•$4H_2O$, 0.4 g $MgSO_4$, 40 mg $FeSO_4$•$7H_2O$, 84 mg $CaCl_2$, 500 µg thiamine•HCl, 0.1 g EDTA, and 100 µg biotin per 1 L. pH was adjusted to 7.2 with KOH. *C. glutamicum* was then grown at 30ºC for 20-24 h (end of growth phase). As for Δ*pks13* mutant of *C. glutamicum*, the cells were grown at 30ºC for 72 h. The shaking speed was 150 rpm. For MV induction, each of MMC and penicillin was added to MM-1 medium to final concentrations of 100 ng mL^{-1} MMC and 0.4 U mL^{-1}, respectively, in early exponential phase. After preincubation in LB medium at 37ºC for 72 h and washing cells, *M. smegmatis* was inoculated into 100 mL of minimum medium (MM-2) consisting 0.7% (v/v) glycerol, 1 g KH_2PO_4, 2.5 g Na_2HPO_4, 0.5 g asparagine, 50 mg ferric ammonium citrate, 0.5 g $MgSO_4$•$7H_2O$, 0.5 mg $CaCl_2$, 0.1 mg $ZnSO_4$ and 200 µg biotin per 1 L. pH was adjusted to 7.0 with KOH. *M. smegmatis* was then grown at 37ºC for 9 days. The shaking speed was 90 rpm. For MV induction, each of MMC and penicillin was added to MM-2 medium to final concentrations of 100 ng mL^{-1} MMC and 0.4 U mL^{-1}, respectively, after 24 h incubation (early exponential phase). *R. equi* was preincubated in LB medium at 37ºC for 24 h and inoculated into 50 mL of fresh LB medium. *R. equi* was then grown at 37ºC for 16 h. The shaking speed was 190 rpm. For MV induction, each of MMC and penicillin was added to the minimum medium to final concentrations of 100 ng mL^{-1} MMC and 0.4 U mL^{-1}, respectively, in early exponential phase.

MV purification and quantification

MVs were isolated and quantified as follows. Culture supernatants were filtered with 0.45 µm pore size PVDF filter (Merck, Darmstadt, Germany). The supernatants were then ultracentrifuged for 1 h at 150,000 × *g*, 4ºC. Pellet was resuspended in 400 µL of 45% iodixanol (Optiprep, AXIS-SHIELD, Dundee, Scotland) in a buffer containing 20 mM HEPES-NaOH (pH 7.0) and 0.85% NaCl. This solution was placed at the base of a 4 mL tube, and then 400 µL of 40, 35, 30, 25, 20, 15 and 10% iodixanol solutions were layered on the top of the suspended solution to make density gradient in the tube. The tube was immediately ultracentrifuged for 3 h at 10,0000 × *g*, 4 ºC. After the ultracentrifugation, a fraction (400-500 µL) containing purified MVs, which appeared as a band in upper middle of the tube, were collected and then washed with 20 mM HEPES-NaOH (pH 7.0) and 0.85% NaCl. Pellet was resuspended in the same buffer, and this solution was used for further analyses. MVs were quantified using the fluorescent dye FM4-64.

Size distributions and particle numbers of MVs

Size distributions and particle numbers of MVs were analysed by Nanosight NS300 (Nanosight Ltd., Malvern, United Kingdom). After washing with 2 mL of H_2O, diluted MV solution was injected into the apparatus. Flow pass was equilibrated with 1.0 mL of the MV solution before analyses. Measurement was performed for three times, and size distributions and particle numbers were analysed based on these data.

Microscopy

Confocal laser scanning microscopy (CLSM) was performed with LSM780 with Airyscan detector (Carl Zeiss, Oberkochen, Germany). For CLSM, bacteria were put on 0.8% agarose pad after staining with 2.5 μg mL^{-1} FM4-64 or 3 μM acridine orange 10-nonyl bromide. Peptidoglycan was stained by culturing bacteria in MM-1 medium containing 10 μM HADA followed by washing cells with fresh MM-1 medium. For live-cell imaging, bacteria were put on MM-1 containing 0.8% agarose, 2.5 μg mL^{-1} FM4-64, 5 μM SYTOX green and 0.1 or 100 μg L^{-1} biotin after staining cells with 2.5 μg mL^{-1} FM4-64. In total, 400 ng mL^{-1} MMC or 0.4 U mL^{-1} was added to the above medium when required. The cells were then incubated and observed at 30ºC.

For transmission electron microscopy (TEM), purified MVs were attached to thin carbon film-coated TEM grids (ALLIANCE Biosystems, Osaka, Japan) and washed with H_2O. MVs were then visualised by negative staining. For TEM inspection of ultra-thin sections of bacterial cells, cells were fixed with a solution containing 100 mM phosphate buffer (pH 7.0) and 2% glutaraldehyde. After the first fixation, the cells were washed twice with 0.1 M phosphate buffer and then further fixed with 2% OsO_4. After the second fixation, the cells were dehydrated with 50%, 70% and 100% ethanol sequentially. The cell suspension was replaced with propylene oxide solution and then embedded in Epon 812. The samples were sliced by ultramicrotome and the resulting ultra-thin sections were visualised with uranyl acetate and lead-containing staining solution.

Quick-freeze, deep-etch electron microscopy

Samples were treated as previously reported (Tulum et al, 2019). MVs were collected by centrifugation at 150,000 × *g*, at 4 ºC for 60 min and suspended in water. The sample suspension was mixed with a slurry that included mica flakes, placed on a rabbit lung slab, and frozen by a metal contact method with CryoPress (Valiant Instruments, St. Louis, MO, USA) cooled by liquid helium. The slurry was used to retain an appropriate amount of water before freezing unless stated in the figure legend. 80% glycerol instead of water when observing the inner leaflet of the lipid bilayer. The specimens were fractured and etched for 15 min at −104°C, in a JFDV freeze-etching device (JEOL Ltd., Akishima,Japan). The exposed MVs were rotary-shadowed by platinum at an angle of 20 degrees to be 2 nm in thickness and backed with carbon. Replicas were floated off on full-strength hydrofluoric acid, rinsed in water, cleaned with a commercial bleach, rinsed in water and picked up onto copper grids as

described. Replica specimens were observed by a JEM 1010 transmission electron microscope (JEOL, Tokyo, Japan) at 80 kV equipped with a FastScan-F214 (T) CCD camera (TVIPS, Gauting, Germany).

Quantification of extracellular double-stranded DNA

Bacteria were grown as described in Culture conditions and removed by centrifugation (7,000 *g* × 10 min). Double-stranded DNA in the resulting supernatant was then quantified using PicoGreen (Life Technologies, Carlsbad, CA, USA). DNA concentration was calculated based on the standard curve obtained from the standard DNA.

Chromatography and mass spectrometry

Liquid chromatography/mass spectrometry (LC/MS) analyses were carried out using Nexera X2 system and an LCMS-8050 (Shimadzu, Kyoto, Japan) equipped with a Kinetex EVO C18 2.6 µm 150 mm × 2.1 mm (PHENOMENEX, Torrance, CA, USA), ZIC-HILIC 3.5 µm 150 mm × 2.1 mm (Merck, Darmstadt, Germany) or TSK-gel ODS-120H 3 µm 2.0 × 150 mm (Tosoh Co., Ltd., Tokyo, Japan). Lipids were analysed using the following conditions: Kinetex EVO C18 column; flow rate, 0.26 mL min^{-1}; temperature, 40ºC; solvent A, H_2O:acetonitrile = 40:60 (v/v) and 10 mM ammonium formate; and solvent B, acetonitrile:2-propanol = 10:90 (v/v) and 10 mM ammonium formate. After column equilibration with 100% solvent A, 2 µl of a sample was injected into the column. The samples were eluted from the column by the following gradient: 0-20 min, 0-100% solvent B; 20-30 min, 100% solvent B; 30-35 min, 0% solvent B. Mycolic acid esters were detected by LC/MS/MS as follows. Mycolic acid esters were detected as $[M+NH_4]^+$ adduct in the positive ion mode and their structures were confirmed by MS/MS analysis with a collision energy -30 or -40 eV.

Amino acids were analysed using the following conditions: ZIC-HILIC column; flow rate, 0.2 mL min^{-1}; temperature, 40ºC; solvent A, H_2O:acetonitrile:formate = 98:1:1 (v/v); and solvent B, H_2O:acetonitrile:formate = 1:98:1 (v/v). After column equilibration with 95% solvent B, 2 µl of a sample was injected into the column. The samples were eluted from the column by the following gradient: 0-1 min, 95% solvent B; 1-10 min, 95-5% solvent B; 10-14 min, 5% solvent B; 14-21 min, 95% solvent B. Concentrations of the samples were calculated based on the standard curve obtained from the standard solution. Amino acids were detected by multiple reaction monitoring (MRM) mode as follows. Alanine (m/z = 89.9 $[M+H]^+$), glutamate (m/z = 148.2 $[M+H]^+$) and *meso*-diaminopimelic acid (m/z = 191.0 $[M+H]^+$) were fragmented into product ions which exhibit m/z values of 44.1, 84.2 and 128.1, respectively, and detected. Collision energies were -13.0 eV, -18.0 eV and -15.0 eV, respectively.

Sugars were analysed using the following conditions: TSK-gel ODS column; flow rate, 0.2 mL min^{-1}; temperature, 40ºC; solvent A, H_2O:acetonitrile:formate = 98:1:1 (v/v) and 5 mM ammonium acetate; and solvent B, H_2O:acetonitrile:formate = 1:98:1 (v/v). After column equilibration with 100% solvent A, 2 µl of a sample was injected into the column. The samples were eluted from the column by the

following gradient: 0-20 min, 0-100% solvent B; 20-25 min, 0% solvent B. Sugars were derivatized (described below) and detected by MRM mode as follows. Arabinose (*m/z* = 481.1 $[M+H]^+$), *N*-acetylglucosamine (*m/z* = 510.1 $[M+H]^+$) and galactose (*m/z* = 511.3 $[M+H]^+$) were fragmented into a product ion which exhibits *m/z* value of 175.2 and detected. Collision energies were -30.0 eV, -32.0 eV and -29.0 eV, respectively.

Gas chromatography/mass spectrometry (GC/MS) analyses were carried out using GC2010 and GCMS-QP2010 (Shimadzu, Japan) equipped with FFAP silica capillary column 0.25 mm × 25 m × 0.25 μm (QUADREX Corp., Woodbridge, CT, USA). Fatty acids including mycolic acids were separated and detected under the following conditions: injector temperature, 320ºC; the flow rate of carrier gas (helium), 24 mL min^{-1}. The sample were eluted from the column by the following temperature gradient: 0-2 min, 80ºC; 2-9 min, 80-220ºC; 9-34 min, 320ºC.

Extracted lipids were separated by thin layer chromatography (TLC) as follows. Samples were spotted onto a silica gel plate and then separated using chloroform:methanol:H_2O = 65:25:4 (v/v, for corynomycolic acid esters and polar lipids), chloroform: hexane: methanol: acetate = 50:30:10:5 (v/v, for phosphatidylglycerols and cardiolipins) and toluene: chloroform: acetone = 7:2:1 (v/v, for diacylglycerols) as the solvents. Based on a previous study (Bansal-Mutalik et al, 2011), L-α-phosphatidyl-DL-glycerol (distearoyl), phophatidylinositol, cardiolipin from bovine heart and 1,2-dipalmitoyl-*rac*-glycerol were used as standards. Silica gel plate was then dried and sprayed with 0.01% (w/v) primuline (in 80% acetone). Fluorescence of the labelled lipids were detected with UV at 365 nm. Obtained images were analysed by ImageJ (Schneider et al, 2012). Amounts of lipids were calculated based on the standard curve of intensities of the above lipid standards.
Matrix-assisted laser desorption/mass spectrometry (MALDI-TOF/MS) was performed using UltrafleXtreme-ETA MALDI-TOF/TOF (Bruker, Billerica, MA, USA). 2-Cyano-3-(4-hydroxyphenyl) acrylic acid was used as a matrix.

Analyses of glutamate and lysine effluxes

Glutamate and lysine were detected and quantified as follows. After culturing *C. glutamicum*, the cells were removed by centrifugation (7,000 *g* × 10 min). Equivalent volume of acetonitrile was added to the resulting supernatant. This solution was filtered (0.45 μm) and then analysed by LC/MS. Cells were lyophilised and then suspended in 50% acetonitrile. After removing debris by centrifugation (20,000 *g* × 5 mi), the resulting supernatant was analysed by LC/MS. The extracted amino acids were separated as described under the above section Chromatography and mass spectrometry, and then detected by MRM mode. Detection conditions for glutamate were as follows: retention time, 8.6 min; a precursor ion, *m/z* = 148.2; a product ion, *m/z* = 84.2; collision energy, -16.0 eV; detection mode, positive. Detection conditions for lysine were as follows: retention time, 10.6 min; a precursor ion, *m/z* = 147.1; a product ion, *m/z* = 84.2; collision energy, -17.0 eV; detection mode, positive. Amounts of

glutamate and lysine were calculated based on the standard curve of the intensities obtained from authentic standards. 1.6 μL mg^{-1} dry cell weight (Nakamura et al, 2007) was used to calculate intracellular glutamate concentrations.

Analyses of lipids and sugars

For GC/MS analyses, lipids were extracted and derivatised as follows. Lyophilised cells (80 mg) and MVs (6~17 mg) were suspended in 500 μL of 10% KOH (in methanol). This solution was incubated at 98ºC for 2 h. After acidification (< pH 2) by adding 6 N HCl, lipids were then extracted by hexane. After removing the solvent, lipids were further incubated and esterified in benzene: methanol: sulfate: = 10:20:1 (v/v) at 98ºC for 2 h. H_2O was then added to the solution, and lipids were extracted by hexane. Covalently bound-mycolic acids of mycolic acid-arabinogalactan-peptidoglycan complexes were extracted and derivatised as described above using purified cell walls (see below). After removing the solvent, 50 μL of pyridine and 100 μL of *N,O*-bis(trimethylsilyl)trifluoroacetamide (BSTFA) were added to the lipids. After incubation at 70ºC for 12 h, the derivatised lipids were subjected to GC/MS analyses.

For LC/MS analyses, lipids are extracted as follows. After cultivation for 12~16 h (the end of exponential phase), cultured cells were harvested by centrifugation and washed with 0.85% NaCl. Cells were then suspended in H_2O-saturated 1-butanol. 1-Butanol extraction was previously reported to potentially enrich mycomembrane lipids of mycolic acid-containing bacteria (Klatt et al, 2018; Morita et al, 2005; Patterson et al, 2000). The cells were removed by centrifugation and resuspended in chloroform: methanol = 2:1. The same extraction was then performed using chloroform: methanol: H_2O = 1:2:0.8. Lipids of MV fractions were extracted by chloroform: methanol = 2:1. All extracts were dried, dissolved with the corresponding solvent, and then subjected to LC/MS analyses. In these analyses, we defined the mycomembrane-specific lipids and the inner membrane-specific lipids as the lipids that were detected in either the mycomembrane (1-butanol extract) or the inner membrane extract (total of chloroform/methanol and chloroform/methanol/water extracts).

Membrane lipids in *C. glutamicum* were identified as follows. Based on our results of GC/MS analyses of derivatised fatty acids (Fig. S12), C16-C18 fatty acids and mycolic acids (C32 and C34) were identified to be major acyl chains consisting mycomembrane and inner membrane lipids of *C. gltamicum* under the tested condition. In addition, previous studies (Bansal-Mutalik et al, 2011; Klatt et al, 2018) and our TLC analysis (Fig. 3 and Fig. S15) showed that trehalose dicorynomycolic acids, phosphatidylglycerol, diacylglycerol, phosphatidylinnositols (including the mannoside derivatives) and cardiolipins are major classes of lipids consisting the membranes. Considering these observations, on LC/MS, we scanned the major membrane lipids in each of the extracts using the theoretical *m/z* values that were calculated from the possible structures of the lipids containing C32-C34 corynomycolic acids or C16-C18 fatty acids

(Hoischen et al, 1990; Klatt et al, 2018). From the result of this analysis, candidate molecules that might belong to each class of lipids were listed. The structures of the candidate lipids were then determined by LC/MS/MS analysis based on *m/z* values of the fragments including the fatty acid moieties and the polar head group moieties. Profiles of the identified lipids of *C. glutamicum* was consistent with those in the previous studies (Cox et al, 2009; Hsu et al, 2001, 2007; Kind et al, 2013; Minkler et al, 2010; Mishra et al, 2009). The characteristics of the fragment patterns of the identified lipids are summarised in Fig. S16. Representative data of LC/MS/MS analysis and the catalogue of the identified lipids are shown in Fig. S16 and S17. Ion intensities of the detected lipids in the scanning mode on LC/MS are shown in Fig. S18.

Cell walls were purified from cells and MVs as follows. *C. glutamicum* cells were disrupted by sonication and centrifuged (5,000 $g \times 30$ min and 20,000 $g \times 30$ min). 4% (w/v) sodium dodecyl sulfate was added to the pellets and the resulting solutions were incubated at 100ºC for 40 min. 10% tween 40 was added to the purified MVs and the resulting solutions were incubated at room temperature for 40 min. After incubation, these solutions were centrifuged (20,000 $g \times 30$ min at 20ºC) and washed with H_2O. The resulting pellets were lyophilised and stored at -20ºC. For LC/MS analyses, the lyophilised cell walls were hydrolysed by 6 N HCl at 100ºC for 3 h. These solutions were then dried and subjected to LC/MS analyses for amino acids or further derivatisation. Dried cell wall components were dissolved with 10 µL of H_2O. 15 µL of 0.5 M NaOH and 25 µL of 0.5 M 3-methyl-1-phenyl-5-pyrazolone (PMP) were then added to the solutions. After incubation at 70ºC for 2 h, 20 µL of 0.5 N HCl was added to these solutions. The resulting solutions were subjected to LC/MS analyses for PMP-derivatised sugars. Amino acids and sugar derivatives were detected by MRM mode. The amounts of amino acids and sugars were quantified based on the standard curve obtained from intensities of authentic standards.

Identification of proteins

Proteins were identified as follows. Cells were disrupted by sonication and the lysates were used for further experiment. The cell lysate and purified MVs solutions were mixed with an equivalent volume of a sample buffer containing 0.1 M Tris-HCl (pH 6.8), 20% (v/v) glycerol, 4% (w/v) SDS, 5% (v/v) 2-mercaptoethanol and 0.2% (w/v) bromophenol blue, and incubated at 98ºC for 5 min. After incubation, these solutions were applied to a gel and then subjected to an electrophoresis. Stacking gel contained 0.15 mL of 30% (w/v) acrylamide/bis mixed solutions (Nacalai Tesque Co., Inc., Kyoto, Japan), 0.25 mL of 1.5 M Tris-HCl (pH 8.8), 10 µL of 10% (w/v) ammonium persulfate and 2 µL of tetramethylethylenediamine per 1 mL. Running gel contained 1.6 mL of 30% acrylamide/bis mixed solutions, 1 mL of 1.5 M Tris-HCl (pH 8.8), 20 µL of 10% (w/v) ammonium persulfate and 4 µL of tetramethylethylenediamine per 4 mL. After the electrophoresis, bands derived from the proteins were stained with Coomassie brilliant blue and then cut out from the gel. Isolated proteins were degraded by trypsin, and then carbamidomethyl group was added to -SH group of cysteine residues of the peptides. The resulting peptides were analysed by MALDI-TOF/MS. Obtained mass spectra derived from these

peptides were analysed by MASCOT database search (Matrix Science Ltd., London, United Kingdom). Proteins that were derived from *C. glutamicum* ATCC13032 and showed the highest MASCOT scores (above significance threshold) were assigned to the bands.

Supplemental References

Armenteros, J. J. A., Tsirigos, K. D., Sønderby, C. K., Petersen, T., N., Winther, O., Brunak, S., von Heijne, G., and Nielsen, H. (2019). SignalP 5.0 improves signal peptide predictions using deep neural networks. Nat. Biotechnol. *37*, 420-423.

Bansal-Mutalik, R., and Nikaido, H. (2011). Quantitative lipid composition of cell envelopes of *Corynebacterium glutamicum* elucidated through reverse micelle extraction. Proc. Natl. Acad. Sci. USA *108*, 15360-15365.

Brennan, P. J., and Nikaido, H. (1995). The envelope of mycobacteria. Annual Reviews of Biochemistry *64,* 29-63.

Bukovski, A. (2013). Cell envelope of Corynebacteria: structure and influence on pathogenicity. ISRN Microbiol. 2013:935736.

Cox, D., Fox, L., Tian, R., Bardet, W., Skaley, M., Mojsilovic, D., Gumperz, J., and Hildebrand, W. (2009). Determination of cellular lipids bound to human CD1d molecules. PLoS One 4:e5325.

Hashimoto, K., Kawasaki, H., Akazawa, K., Nakamura, J., Asakura, Y., Kudo, T., Sakuradani, E., Shimizu, S., and Nakamatsu, T. (2006). Changes in composition and content of mycolic acids in glutamate-overproducing *Corynebacterium glutamicum*. Biosci. Biotechnol. Biochem. *70*, 22-30.

Hoischen, C., and Kräman, R. (1990). Membrane alteration in necessary but not sufficient for effective glutamate secretion in *Corynebacterium glutamicum*. J. Bacteriol. *172*, 3409-3416.

Hsu, F. F., Turk, J., Owens, E. R., and Russell, D. G. (2007). Structural characterization of phosphatidyl-*myo*-inositol mannosides from *Mycobacterium bovis* Bacillus Calmette Guérin by multiple-stage quadrupole ion-trap mass spectrometry with electrospray ionization. I. PIMs and lyso-PIMs. J. Am. Soc. Mass Spectrom*.* *18*, 466-478.

Hsu, F. F., and Turk, J. (2001). Studies on phosphatidylglycerol with triple quadrupole tandem mass spectrometry with electrospray ionization: fragmentation processes and structural characterization. J. Am. Soc. Mass Spectrom*.* *12*, 1036-1043.

Kind, T., Liu, K. H., Lee, D. Y., DeFelice, B., Meissen, J. K., and Fiehn, O. (2013). LipidBlast *in silico* tandem mass spectrometry database for lipid identification. Nature Methods *10*, 755-758.

Klatt, S., Brammananth, R., O'Callaghan, S., Kouremenos, K. A., Tull, D., Crellin, P. K., Coppel, R. L., and McConville, M. J. (2018). Identification of novel lipid modifications and intermembrane dynamics in *Corynebacterium glutamicum* using high-resolution mass spectrometry. J. Lipid Res*.* *59*, 1190-1204.

Minkler, P. E., and Hoppel, C. L. (2010). Separation and characterization of cardiolipin molecular species by reverse-phase ion pair high-performance liquid chromatography-mass spectrometry. J. Lipid Res. *51*, 856-865.

Mishra, A. K., Batt, S., Krumbach, K., Eggeling, L., and Besra, G. S. (2009). Characterization of the *Corynebacterium glutamicum* Δ*pimB'* Δ*mgtA* double deletion mutant and the role of *Mycobacterium tuberculosis* orthologues Rv2188c and Rv0557 in glycolipid biosynthesis. J. Bacteriol. *191*, 4465-4472.
Mitaku, S., Hirokawa, T., and Tsuji, T. (2002). Amphiphilicity index of polar amino acids as an aid in the characterization of amino acid preference at membrane-water interfaces. Bioinformatics *18*, 608-616.
Morita, Y. S., Velasquez, R., Taig, E., Waller, R. F., Patterson, J. H., Tull, D., Williams, S. J., Billman-Jacobe, H., and McConville, M. J. (2005). Compartmentalization of lipid biosynthesis in Mycobacteria. J. Biol. Chem. *280*, 21645-21652.
Nakamura, J., Hirano, S., Ito, H., and Wachi, M. (2007). Mutations of the *Corynebacterium glutamicum* NCgl1221 gene, encoding a mechanosensitive channel homolog, induce L-glutamic acid production. Appl. Environ. Microbiol. *73*, 4491-4498.
Patterson, J. H., McConville, M. J., Haites, R. E., Coppel, R. L., and Billman-Jacobe, H. (2000). Identification of a methyltransferase from *Mycobacterium smegmatis* involved in glycopeptidolipid synthesis. J. Biol. Chem. *275*, 24900-24906.
Portevin, D., Sousa-D'Auria, C. D., Houssin, C., Grimaldi, C., Chami, M., Daffé, M., and Guilhot, C. (2004). A polyketide synthase catalyzes the last condensation step of mycolic acid biosynthesis in mycobacteria and related organisms. Proc. Natl. Acad. Sci. USA *101*, 314-319.
Schneider, C.A., Rasband, W.S., and Eliceiri, K.W. (2012). NIH Image to ImageJ: 25 years of image analysis. Nat. Methods *9*, 671-675.
Tulum I., Tahara, Y. O., and Miyata, M. (2019). Peptidoglycan layer and disruption processes in *Bacillus subtilis* cells visualized using quick-freeze, deep-etch electron microscopy. Microscopy *68*, 441-449.
van der Rest, M. E., Lange, C., and Molenaar, D. (1999). A heat shock following electroporation induces highly efficient transformation of *Corynebacterium glutamicum* with xenogeneic plasmid DNA. Appl. Microbiol. Biotechnol. *52*, 541-545.
